# RNA 2'-O-methylation promotes persistent R-loop formation and AID-mediated IgH class switch recombination

**DOI:** 10.1186/s12915-024-01947-5

**Published:** 2024-07-08

**Authors:** Muzaffer Ahmad Kassab, Yibin Chen, Xin Wang, Bo He, Eric J. Brown, Xiaochun Yu

**Affiliations:** 1 Department of Cancer Genetics and Epigenetics, Beckman Research Institute, City of Hope Medical Center, Duarte, CA 91010 USA; 2 Present address: Department of Cancer Biology, Perelman School of Medicine, University of Pennsylvania, Philadelphia, PA 19104 USA; 3 Present address: Therapeutics Discovery Division, The University of Texas MD Anderson Cancer Center, Houston, TX 77054 USA; 4 Present address: Division of Cellular and Developmental Biology, Department of Molecular and Cell Biology, University of California, Berkeley, CA 94705 USA; 5 Present address: Westlake University, Hangzhou, Zhejiang P. R. China

**Keywords:** R-loop, Class switching recombination (CSR), Activation-induced cytidine deaminase (AID), 2'-O-methylation (2'-OMe)

## Abstract

**Background:**

RNA–DNA hybrids or R-loops are associated with deleterious genomic instability and protective immunoglobulin class switch recombination (CSR). However, the underlying phenomenon regulating the two contrasting functions of R-loops is unknown. Notably, the underlying mechanism that protects R-loops from classic RNase H-mediated digestion thereby promoting persistence of CSR-associated R-loops during CSR remains elusive.

**Results:**

Here, we report that during CSR, R-loops formed at the immunoglobulin heavy (IgH) chain are modified by ribose 2′-O-methylation (2′-OMe). Moreover, we find that 2′-O-methyltransferase fibrillarin (FBL) interacts with activation-induced cytidine deaminase (AID) associated snoRNA aSNORD1C to facilitate the 2′-OMe. Moreover, deleting AID C-terminal tail impairs its association with aSNORD1C and FBL. Disrupting FBL, AID or aSNORD1C expression severely impairs 2′-OMe, R-loop stability and CSR. Surprisingly, FBL, AID’s interaction partner and aSNORD1C promoted AID targeting to the IgH locus.

**Conclusion:**

Taken together, our results suggest that 2′-OMe stabilizes IgH-associated R-loops to enable productive CSR. These results would shed light on AID-mediated CSR and explain the mechanism of R-loop-associated genomic instability.

**Supplementary Information:**

The online version contains supplementary material available at 10.1186/s12915-024-01947-5.

## Background

During immunoglobulin class switch recombination (CSR), immunoglobulin isotype is changed from IgM (Cμ) to IgG (γ), IgE (ε), or IgA (α). CSR is accomplished by the genetic rearrangement in the constant region of the immunoglobulin heavy (IgH) chain. The IgH chain constant region harbors the exons for the different isotypes in distinct clusters on chromosome 14 and 12 in humans and mouse respectively. Each exon cluster is flanked by an upstream regulatory DNA sequence known as the switch region (S) [1, 2]. Transcription at the S region promotes the formation of RNA–DNA hybrids (R-loops) thereby exposing the non-template strand [3–6].

CSR is regulated by the recruitment of activation-induced cytidine deaminase (AID) to switch regions wherein AID accesses the non-template strand and deaminate dC into dU, which is further processed and leads to the breakage of DNA [1, 7–9]. The switch region DNA breaks induce the deletion of intervening genomic DNA in the IgH loci and joining of an upstream Sμ region with a downstream S-region [1, 10]. The switch regions are rich in repetitive GC nucleotides (G-rich) and transcriptional activity at these regions promotes R-loop formation and these R-loops aid in CSR [6, 11, 12]. Preventing R-loop formation by deleting or inverting the endogenous S region impairs CSR that could be restored by replacing the deleted S region with an artificial DNA sequence capable of forming R-loops [13, 14]. R-loop requirement in CSR was additionally characterized by digesting them using RNase H, RNase H cleaves the RNA component in the R-loop, and therapy impedes the formation of stable R-loops [15, 16]. However, one of the studies observed that RNase H treatment decreased CSR by 50% and proposed that R-loops could be involved in a step downstream to the initial AID deamination step [15]. An independent study reported RNase H treatment led to an increase in somatic hypermutation in the non-template strand without affecting CSR [16]. Thus, to gain a clear insight into the role of R-loops in CSR, it was absolutely essential to know the chemical nature of these R-loops.

Within the IgH locus, the G-rich sequences are distributed across an array of conserved AGCT motifs, and these sequence motifs act as hotspots of AID-induced mutations [17–19]. During transcription, the G-rich sequences on the non-template DNA [20] and the transcribed RNA [21] fold into a highly stable, non-canonical four-stranded secondary structure known as G-quadruplexes (G4) [22]. RNA helicase DDX1 binds to the G4 RNA and resolves them into R-loop forming RNAs [20]. The resulting R-loops promotes formation of G4 structures in the displaced DNA and the G4 in turn stabilizes the R-loops in a positive feedback loop [6, 23, 24]. The G4s also provide a preferential docking site to AID and promote its cooperative oligomerization on the IgH locus [20, 25]. Moreover, the newly formed non-canonical R-loops and G4s stall transcription of the region as evidenced by a buildup of stalled RNA pol II markers SPT5 and RNA exosome [26]. AID binds to Spt5 and RNA exosome on the stalled RNA polymerase II that promotes additional AID buildup and its targeting to both template and non-template DNA [26–29]. RNA exosome removes the R-loop-associated RNAs and this removal is further assisted by RNA processing proteins SETX and RNase H2 [8, 28, 30, 31]. Recent studies suggest that HNRNPU binds to the S region G4s and R-loops and prevents R-loop accumulation and extensive DNA breaks thereby facilitates the recruitment of repair proteins required for an optimal CSR [19].

R-loops are also frequently formed at non-IgH loci during transcription when a nascent RNA stably hybridizes to its template DNA, leaving the non-template tract in a single-stranded conformation [32, 33]. Unfortunately, R-loops formed at non-IgH loci are highly unstable structures that can cause genomic instability. Consequently, R-loops are rapidly removed by RNase H enzymes to maintain genomic stability [32, 34, 35]. However, the underlying molecular mechanisms responsible for the selective persistence of R-loops at the IgH locus are unclear.

Interestingly, posttranscriptional modifications on RNA are known to modulate RNA stability and function [36]. One of the most prevalent RNA posttranscriptional modifications is 2′-O-methylation (2'-OMe) of the ribose sugar. Among several enzymes known to catalyze 2'-OMe, one of the most abundant 2'-OMe transferases is fibrillarin (FBL) [37] which catalyzes 2'-OMe on the rRNA. FBL uses C/D box snoRNAs as guide RNAs during 2'-OMe of the target rRNAs. The snoRNAs base pair with the rRNA and FBL deposits 2'-OMe on the target rRNA. 2'-OMe modulates the conformation of the ribose sugar in the rRNA thereby affecting its structure, function, and stability [38, 39].

Although the role of R-loops in CSR is heavily investigated [5, 14], the cellular mechanism responsible for their selective retention during this process is elusive. Here, we report that R-loops at IgH locus contain 2′-OMe, which stabilizes them and prevents their degradation. We identified FBL as the 2′-O-methyltransferase required for catalyzing 2'-OMe on these R-loops. Moreover, we found that FBL interacts with AID-associated snoRNA aSNORD1C and facilities R-loop formation and CSR. Although a single 2′-OMe site may not be a dominant feature responsible for R-loop stability, RNA 2′-OMe is important for proper cellular function of the global RNAs. The 2′-OMe changes conformation of the RNA, protects the RNA from degradation [40], and modulates the association of cellular proteins with the modified RNA [41–44].

In addition, previous studies suggest that the C-terminus of AID has indispensable function in CSR and loss of AID C-terminus abolishes CSR without altering somatic hypermutation function of AID [45, 46]. Surprisingly, we observed a drastic decrease in AID targeting upon aSNORD1C and FBL alteration. We propose that the RNA partners (aSNORD1C) of AID may be required for channelizing AID towards CSR, and aSNORD1C could function as a potential RNA required for these CSR-dependent functions. The RNA partners may function in guiding AID to the switch regions and promote AID recruitment specifically to the switch regions thereby preventing global deamination of the DNA.

## Results

### FBL modifies IgH-associated germline transcripts

**Fig. 1:**
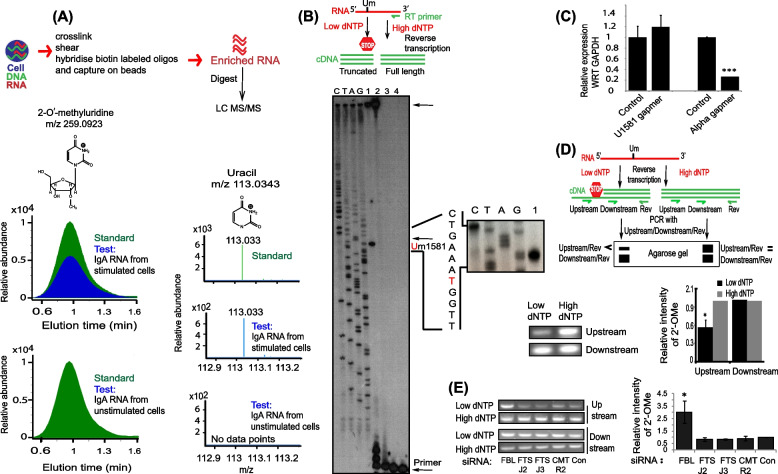
FBL modifies IgH germline transcripts. **A** Top panel shows flowchart of the method employed to enrich RNA. The bottom diagram shows LC-MS/MS analysis of enriched Sα RNA from CH12F3 cells. The cells were unstimulated interaction partner or stimulated with CIT*,* and the RNA was enriched using the ChIRP protocol. The extracted ion chromatogram (EIC) of *m/z* 113.03 (uridine) was derived from the MS/MS scan of 2′-O-methyluridine (Um) at *m/z* 259.09. Um spectral peaks were present in the RNA obtained from stimulated CH12F3 cells and absent in RNA isolated from unstimulated cells (test: blue color). The chemical structure and the mass spectral properties of chemically synthesized Um (standard: green color) eluted at 1 min are shown and overlapped with the test sample. **B** Top panel shows schematic representation of primer extension assay. Reverse transcription of a target RNA containing 2′-OMe U (Um) is performed at either low or high concentrations of dNTP (low dNTP: 0.004 mM; high dNTP: 0.5 mM). Under low dNTP concentrations, majority of RT product will terminate at the Um site resulting in the formation of strong truncated cDNA band in the gel. However, under high dNTP concentrations, full-length cDNA will be formed. The bottom gel image shows primer extension of enriched IgA RNA from stimulated and unstimulated cells. The position of Um1581, the primer used for RT and the full-length cDNA product are labeled. 1: RT of RNA from stimulated CH12F3 cells at low dNTP; 2: RT of RNA from stimulated CH12F3 cells at high dNTP; 3: RT of RNA from unstimulated CH12F3 cells at low dNTP concentrations; 4: RT of RNA from unstimulated CH12F3 cells at high dNTP. A sequencing ladder (CTAG) was run alongside the cDNA products. A zoomed-in picture around the target site is show on the left side of the figure. The nucleotides around the RNA target site (U1581) are shown on the left side and the target base in the cDNA is highlighted in red color. **C** Quantification of RNA after ASO knockdown. U1581 RNA was unaltered while IgA RNA was efficiently suppressed after ASO transfection in stimulated CH12F3 cells. **D** Top panel shows schematic representation of an RTLP assay. Reverse transcription of a target RNA containing 2′-OMe U (Um) is performed at either low or high concentrations of dNTP. Under low dNTP concentrations, majority of RT product will terminate at the Um site resulting in the formation of a shorter cDNA. However, under high dNTP concentrations, full-length cDNA will be formed. The cDNA formed under the two conditions of dNTP is then PCR amplified with two forward (Upstream is a forward primer upstream to the Um site and Downstream is a forward primer downstream to the Um site) and one reverse primer. Because, Upstream/Rev amplifies full-length cDNA, low-intensity PCR product will be formed from low dNTP reverse transcription reaction in comparison to the PCR product obtained from high dNTP concentrations. However, the Downstream/Rev primer amplifies downstream to the Um site, thus the PCR product formation efficiency will be unaltered under the two dNTP concentrations. Um: 2′-OMe uridine in an RNA, Upstream: forward primer upstream to the Um site, Downstream: reverse primer downstream to the Um site. The bottom panel shows the intensity of cDNA bands obtained after RTLP analysis of RNA. Total cellular R-loops were isolated from CH12F3 cells and gene specific primers were used for quantification of 2′-OMe in the RNA within IgA R-loops. Bottom panel shows the mean levels of 2′-OMe in the R-loops ± the standard deviation from three independent experiments, *p < 0.05. **E** The levels of Um1581, as determined by RTLP after the siRNA-mediated knockdown of FBL, FTSJ2, FTSJ3, CMTR2, or treatment with control (Con) siRNA. (Right) The error bars show the mean levels of 2′-OMe at Um1581 ± the standard deviation from three independent biological replicates, *p < 0.05. The Statistical significance was determined using a two-tailed Student’s *t*-test and *P*-values less than 0.05 were considered significant. The data represents three independent experiments and error bars represent ± standard error of the mean

Cellular R-loops are rapidly degraded by RNase H enzymes [47]. To explore the mechanisms that would enable the selective retention of IgH-associated beneficial R-loops, we stimulated mouse B cell line CH12F3 with anti-CD40, IL-4, and TGF-β (hereafter termed as CIT) to induce CSR from IgM to IgA. To search for possible modifications on the transcribed RNA, we purified germline transcript α (Iα RNA) from the stimulated cells using chromatin isolation by RNA purification (ChIRP) assays. The purified RNA was subsequently digested into nucleosides and subjected to tandem liquid chromatography mass spectrometry (LC–MS/MS) (Fig. 1A). Interestingly, we observed a peak that corresponded to 2′-O-methyluridine (Um), indicating the presence of Um in the Iα RNA. We overlapped the peaks from a synthetic Um as well as the Um derived from the IgA RNA and the peaks showed a perfect overlap. We did not observe Um modifications in the RNA isolated from unstimulated CH12F3 cells which served as a negative control. We next performed ChIRP and mass spectrometry (LC–MS/MS) analysis of germline transcript μ (Iμ RNA) from stimulated CH12F3 cells and obtained similar results (Additional file 1: Fig. S1A). This suggests that presence of Um modification is not an exclusive feature of Iα RNA but can be observed in other RNAs like Iμ.

In order to directly map the position of 2′-OMe (Nm) modifications on the Iα RNA, we designed gene specific primers for Iα RNA and subjected the purified RNA to primer extension at low dNTP concentration. Primer extension is a standard assay for detecting 2′-OMe bases. The assay is based on the principle that at low dNTP concentrations, the reverse transcriptase enzyme pauses at the modified bases, generating a truncated cDNA prematurely terminating before the modified nucleotide (Fig. 1B top). We ran the cDNA samples on a sequencing gel and found a strong cDNA band in the switch region of Iα, corresponding to the position of 2′-O-methylated uridine U1581 (nucleotide 1581 of D11468.1, hereafter termed as Um1581) (Fig. 1B bottom). Moreover, the cDNA band was absent in the RNA purified from unstimulated cells or cDNA generating from stimulated RNA at normal dNTP concentrations, suggesting the specificity of this modification to Iα RNA. We next purified and cloned the cDNA formed under low dNTP concentrations and performed Sanger sequencing. Our sequencing results confirmed the primer extension results with the putative Um1581 (Additional file 1: Fig. S1B). Next to demonstrate whether U1581 was indeed 2′-OMe, we performed an LNA gapmer/RNase H mediated digestion of the target site. LNA gapmers are highly potent oligonucleotides (DNA) that are routinely used for knockdown of target RNAs. The gapmer DNA base pairs with the target RNA and the resulting DNA-RNA duplex is degraded by RNase H. However, a DNA–RNA duplex resists RNase H degradation if the RNA within the duplex is modified by 2′-OMe [48, 49]. We designed two LNA probes against U1581 site as well as the IgA constant region. We observed that U1581 region RNA was resistant to RNAse H mediated digestion while IgA RNA was degraded by the LNAs (Fig. 1C). These results suggest that the modification at U1581 is indeed a 2′-OMe. Taken together, these results establish that Iα RNAs are modified by 2'-OMe.

Next, we directly determined the presence of 2′-O-methylation on the R-loop. We isolated R-loops from stimulated CH12F3 cells using DNA–RNA immunoprecipitation (DRIP) assays with S9.6 monoclonal antibodies. The DNA in the enriched R-loops was removed with DNase I digestion and the RNA was isolated. The 2′-O-methylation levels on the isolated RNA were determined using reverse transcription at low dNTP concentration followed by polymerase chain reaction (RTLP) [40]. This method is based on the principle that cDNA synthesis from an RNA template is impaired due to 2′-OMe at a particular site. The cDNA is formed at both low and high dNTP concentrations and quantified by PCR [50] (Fig. 1D top). We observed drastic decrease in PCR product band when cDNA was formed at low dNTP concentration while no such change was observed when the cDNA was generated under high dNTP concentration (Fig. 1D bottom). These results demonstrate that the 2′-O-methylation is present on the R-loop-associated RNA.

Apart from the IgH region, R-loops are formed globally at many loci within the genome. We next aimed to investigate the 2′-O-methylation profile of global R-loops. We stimulated CH12F3 cells and enriched the R-loops using DRIP assays. The DNA in the enriched R-loops was depleted using DNase I and the RNA was purified. The RNA was digested into nucleosides and analyzed by LC–MS/MS. In these assays, we did not observe significant 2′-O-methylation enrichment (Additional file 1: Fig. S1C). These results suggest that 2′-O-methylation may not be a common feature of R-loops. Instead, it may be uniquely present in a set of R-loops such as the R-loops formed at IgH loci.

To identify the 2′-O-methyltransferases regulating the levels of methylation on Um1581, we used siRNAs to knockdown known 2′-O-methyltransferases FBL, FTSJ2, FTSJ3, and CMTR2 (Additional file 1: Fig. S1D). The methyltransferases were knocked down in CIT-stimulated CH12F3 cells and the levels of methylation on Um1581 were determined by RTLP. Interestingly, knockdown of FBL by siRNA was associated with a 2.5-fold increase in the RTLP signal with respect to the control, suggesting a strong decrease in the levels of 2′-OMe at U1581 (Fig. 1E). However, we did not observe any significant change in the levels of Um1581 upon FTSJ2, FTSJ3, or CMTR2 knockdown. These results suggest that FBL mediates 2′-OMe on Iα RNA.

### FBL is required for productive CSR

**Fig. 2:**
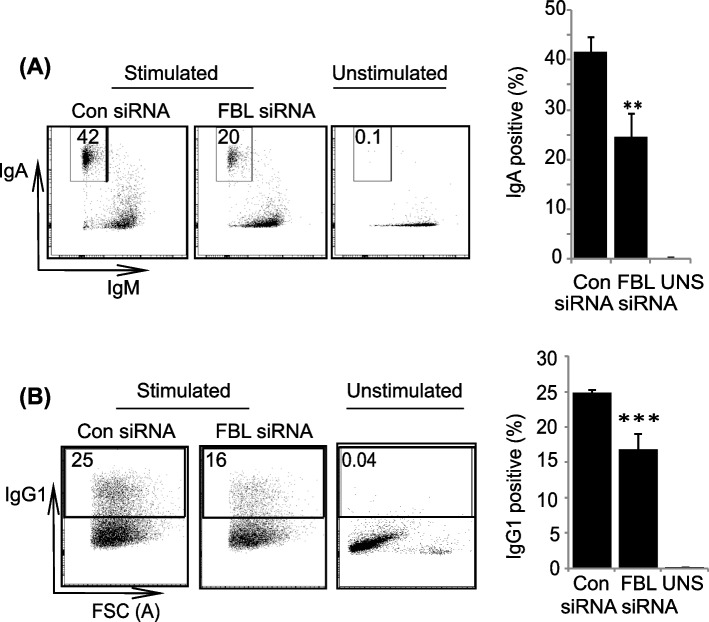
FBL is required for optimal CSR. **A** Flow cytometry analysis of IgM and IgA on the surface of stimulated and unstimulated CH12F3 cells. Stimulated cells were transfected with either FBL siRNA or a control (Con) siRNA and grown for 24 h before CIT stimulation with CIT for 48 h. The percentages of cells switching to IgA are indicated in boxes within each plot. Data are representative of three biological replicates. Two additional experiments confirmed similar results. The right panel shows the mean levels of class switching to IgA in CH12F3 cells after FBL knockdown ± the standard deviation from three independent experiments, **p ≤ 0.01. **B** Flow cytometric analysis of IgG1 on the surface of stimulated and unstimulated splenic B cells. The cells were stimulated with CIL for 24 h. The cells were then transfected with either FBL siRNA or a control (Con) siRNA and grown for 48 h. The percentages of cells switching to IgG1 are indicated in boxes within each plot. Data are representative of three biological replicates. Two additional experiments confirmed similar results. The right panel shows the mean levels of class switching to IgG1 in splenic B cells after FBL knockdown ± the standard deviation from three independent experiments, ***p ≤ 0.001. The statistical significance was determined using a two-tailed Student’s *t*-test and *P*-values less than 0.05 were considered significant. The data represents three independent experiments and error bars represent ± standard error of the mean

AID-mediated antibody formation involves somatic hypermutaion (SHM) and class switching recombination (CSR) in the immunoglobulin locus [4]. During SHM, AID diversifies the immunoglobulin variable region in an R-loop-independent manner [51]. However, R loops promote AID mediated CSR. To determine the contribution of FBL-mediated Um1581 in CSR, we knocked down FBL in CH12F3 cell by siRNAs (Stealth siRNA/Silencer® siRNA pool-Thermo Fisher), pre-validated for specificity and potency. The efficiency of siRNA transfection and knockdown was confirmed by including siRNAs for AID as a positive control (Additional file 1: Figs. S2A-S2B). FBL was knocked down in CH12F3 cells (Additional file 1: Fig. S1D) and the cells were stimulated to undergo CSR to IgA using CIT. We observed that CSR was 52% lower in FBL knockdown cells compared to control siRNA transfected cells (Fig. 2A). To determine the role of FBL in non-IgA CSR, we purified naïve B cells from mouse spleens and stimulated them with anti-CD40, IL-4, and LPS (hereafter termed as CIL) to induce CSR to IgG1. Similar to CSR in CH12F3 cells, we observed significant reduction in IgG1 in FBL knockdown cells than that in control cells (Fig. 2B and S1E). Nevertheless, we did not observe significant changes in cell growth upon siRNA treatment (Additional file 1: Fig. S2C). Furthermore, the primary targets of FBL mediated 2′-OMe are rRNA, wherein 2′-OMe governs an optimal ribosomal structure and function. However, we observed that the reduction of FBL by siRNA to the current level did not alter global cellular translation (Additional file 1: Fig. S2D). Thus, these results suggest that FBL plays an important role during IgA and IgG1 CSR.

### Iα 2'-OMe suppresses R-loop decay

**Fig. 3:**
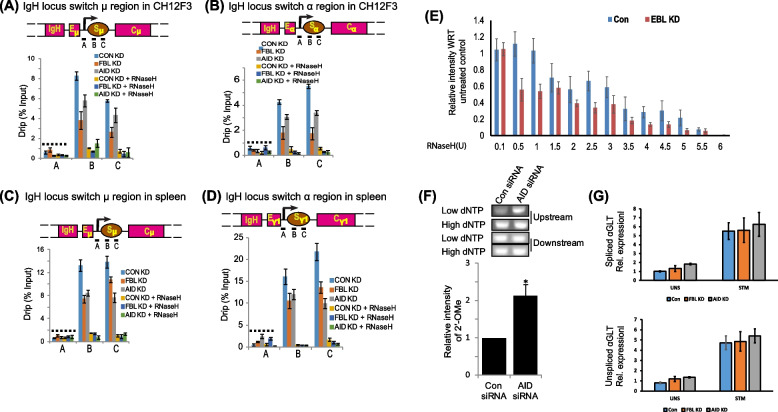
2′-O-Me of germline transcripts prevents R-loop decay. **A** The top panel depicts the schematic representation of the IgM locus, showing the position of the three primers used for quantification of switch RNA-associated R-loops. The bottom panel shows the expression of DNA-RNA hybrids (DRIP) at the IgM locus after FBL and AID knockdown in stimulated CH12F3 cells. One set of samples was treated with RNase H prior to immunoprecipitation using S9.6 antibodies. Pull-down efficiency was calculated using total input DNA as a normalization control. Error bars represent standard deviation of three independent experiments. **B** The top panel depicts the schematic representation of the IgA locus, showing the position of the three primers used for quantification of switch RNA-associated R-loops. The bottom panel shows the expression of DNA-RNA hybrids (DRIP) at the IgA locus after FBL and AID knockdown in activated CH12F3 cells. One set of samples was treated with RNase H prior to immunoprecipitation using S9.6 antibodies. Pull-down efficiency was calculated using total input DNA as a normalization control. Error bars represent standard deviation of three independent experiments. **C** Levels of DNA–RNA hybrids (DRIP) at the IgM locus after FBL and AID knockdown in activated spleen B cells. One set of samples was treated with RNase H prior to immunoprecipitation using S9.6 antibodies. The immunoprecipitation was carried with S9.6 antibody. Pull-down efficiency was calculated using total input DNA as a normalization control. Error bars represent standard deviation of three independent experiments. **D** The top panel shows the schematic representation of IgG1 locus showing the position of three primers used for quantification of switch region-associated R-loops. The bottom panel shows the expression of DNA-RNA hybrids (DRIP) at the IgG1 locus after FBL and AID knockdown in stimulated CH12F3 cells. One set of samples was treated with RNase H prior to immunoprecipitation using S9.6 antibodies. The pull-down efficiency was calculated using total input DNA as a normalization control. (Bottom) The panel represents results from three independent experiments and error bars represent standard deviation of those experiments *p < 0.05. **E** Quantification of switch region-associated R-loops. RNase H was used (0.1–6 U) to digest the R-loops obtained from stimulated CH12F3 cells (Con: wild type, FBL KD: FBL knockdown cells) prior to immunoprecipitation using S9.6 antibodies. The relative levels of R-loops levels normalized to an untreated sample are shown. **F** The top panel shows the levels of Um1581 after AID knockdown determined by RTLP. The bottom panel shows average values and standard deviation from the mean of the PCR product signal intensity of three biological replicates. **G** Analysis of spliced (top panel) and unspliced (bottom panel) IgA germline transcripts in scramble siRNA (con), FBL KD, and AID KD CH12F3 cells as measured by qPCR. The Statistical significance was determined using a two-tailed Student’s *t*-test and *P*-values less than 0.05 were considered significant. The data represents three independent experiments and error bars represent ± standard error (SD) of the mean

The predominant function of switch regions of the germline transcript RNAs is to form R-loops, which contribute to CSR [11, 13, 14]. However, R-loops are routinely subjected to RNase H-mediated degradation. Because 2′-OMe is known to promote RNA stability [52, 53] and prevent RNase H-mediated digestion [48, 49, 54], we investigated whether 2′-OMe stabilizes switch region-associated R-loops during CSR. We evaluated R-loop levels during IgA and IgG1 CSR in stimulated CH12F3 and splenic B cells respectively using DNA–RNA immunoprecipitation (DRIP) assays with S9.6 monoclonal antibodies. Interestingly, we observed that FBL knockdown was accompanied by decrease in the levels of R-loop signal compared to control cells at Sα and Sµ regions (Figs. 3A-B). The dependence of Sµ R-loop levels on FBL expression suggests that non-IgA-associated Nm modifications may be prevalent across switch RNAs. In addition, to our surprise, we also observed lower R-loop signals in the AID knockdown cells than that in control cells, suggesting a possible role for AID in maintaining R-loops at the IgH region. This decreased R-loop abundance upon knockdown was confirmed at earlier time points (24 h) of knockdown (Additional file 1: Fig S3). Similar results were obtained in the Sµ and Sγ1 regions in splenic B cells depleted of FBL or AID (Figs. 3C-D). Additionally, to validate that the R-loop signal was indeed from the switch region-associated R-loops, we treated our samples with RNase H. RNase H treatment drastically reduced the R-loop signals than that in untreated cells, validating the presence of R loops at these regions (Figs. 3A-D). Moreover, no significant enrichment of R-loops was observed at the genomic sites flanking the switch regions (Figs. 3A-D). To further validate that the levels of R-loop are dependent upon 2′-OMe, we performed RNase H titration experiment in CH12F3 cells with or without FBL knockdown. As suspected, we observed that R-loops derived from FBL knockdown cells are more susceptible to RNase H-mediated digestion than those R-loops isolated from the wild-type cells (Fig. 3E). To validate the role of AID in mediating R-loop abundance, we examined the levels of 2′-OMe at Um1581. We observed that siRNA-mediated knockdown of AID was associated with twofold reduction in 2′-OMe at Um1581 (Fig. 3F). To firmly establish the role of FBL and AID on S-region RNA levels, we knocked down these genes using siRNAs and examined the levels of both spliced and unspliced alpha-GLT using qPCR (Fig. 3G). We observed that knockdown of either FBL or AID did not significantly alter the levels of spliced or unspliced alpha-GLT. These results suggest that knockdown of FBL does not alter global S-region RNAs. From our results, we speculate theta FBL is specifically modifying the R-loop-associated S-region RNAs and that this modification favors their accumulation. Collectively, these results suggest that reduced levels of FBL and AID are associated with decrease in R-loop abundance at IgH switch regions.

#### FBL associates with AID during CSR

**Fig. 4:**
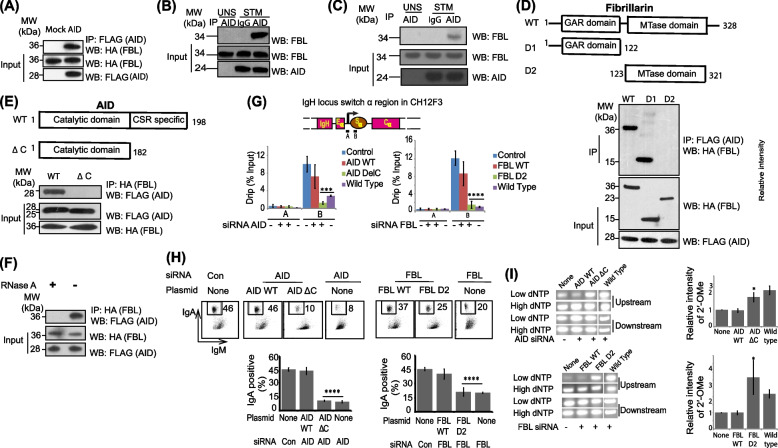
FBL associates with AID during CSR. **A** In-vitro FBL and AID interaction. FLAG-tagged AID and HA-tagged FBL were transfected into HEK293T cells using Lipofectamine 2000 and reversible co-immunoprecipitation (co-IP) was performed on the cell lysates 24 h after Transfection. **B** Reversible co-IP of endogenous AID and FBL in activated CH12F3 cells. CH12F3 cells were unstimulated (UNS) or stimulated (STM) with CIT for 48 h and the cell lysate was analyzed by Western blot. **C** Western blot analysis shows co-IP of endogenous AID and FBL in activated splenic B cells. Purified spleen B cells were unstimulated (UNS) or stimulated with CIL for 48 h and the cell lysate was analyzed by Western blot. **D** The top panel is a schematic representation of FBL, showing the position of the N-terminal GAR domain and C-terminal methyltransferase (MTase) domain. The FBL wild type (WT), FBL deletion constructs D1 (GAR domain), and D2 (MTase domain) were constructed in pCMV-HA-N vectors and transfected into HEK293T cells using Lipofectamine 2000 and co-IP was performed using the indicated antibodies. **E** The top panel is a schematic representation of AID, showing the position of the N-terminal catalytic domain and C-terminal CSR-specific region. AID WT construct (WT), the AID deletion construct, AIDΔC, which lacks the CSR-specific region, was constructed in a pS-Flag-SBP vector and transfected into HEK293T cells using Lipofectamine 2000, and co-IP was performed using the indicated antibodies. **F** RNA mediates the association between AID and FBL. FLAG-tagged AID and HA-tagged FBL were transfected into HEK293T cells using Lipofectamine 2000 and 24 h later, co-immunoprecipitations (co-IPs) was performed on the cell lysates in the presence or absence of RNase A. **G** Ectopic expression of wild-type AID and FBL completely rescued the R-loop levels at the IgA switch locus while AIDΔC and FBL-D2 did not. CH12F3 cells were transiently transfected with respective siRNA and co-transfected with various expressions constructs and grown for 24 h. The cells were CIT stimulated and the R-loops were isolated by DRIP assay. The top panel depicts the schematic representation of the IgA locus, showing the position of the two primers used for quantification of switch RNA-associated R-loops. The bottom panel shows the expression of DNA-RNA hybrids (DRIP) at the IgA locus after FBL and AID knockdown and rescue. Pull-down efficiency was calculated using total input DNA as a normalization control. Error bars represent standard deviation of three independent experiments. **H** Ectopic expression of wild-type AID and FBL rescued CSR to IgA while cells transfected with AIDΔC and FBL-D2 had significantly low percentage of CSR. CH12F3 cells were transiently transfected with respective siRNA and co-transfected with various expressions constructs and grown for 24 h. The cells were CIT stimulated and CSR efficiency was determined using flow cytometry. Bottom: (left) CSR efficiency to IgA in CIT stimulated CH12F3 cells after knockdown followed by complementation with various constructs ± the standard deviation from three independent experiments, **p ≤ 0.01. (Right) CSR efficiency to IgA in CIT stimulated CH12F3 cells after FBL knockdown followed by complementation with various constructs ± the standard deviation from three independent experiments, *p < 0.05. **I** Ectopic expression of wild-type AID and FBL rescued the levels 2′-OMe at Um1581 while cells transfected with AIDΔC and FBL-D2 failed to do so. CH12F3 cells were transiently transfected with respective siRNA and co-transfected with various expressions constructs wild-type (WT) AID or AID deletion construct-AID ΔC and grown for 24 h. The cells were CIT stimulated and 2′-OMe levels were determined using RTLP assays. (Right) RTLP signals obtained after endogenous knockdown AID/FBL and CIT-stimulated CH12F3 cells followed by complementation with various constructs mean ± standard deviation of PCR product from three biological replicates of the RTLP experiments *p < 0.05. The statistical significance was determined using a two-tailed Student’s *t*-test and *P*-values less than 0.05 were considered significant. The data represents three independent experiments and error bars represent ± standard error of the mean

Next, we further examined the molecular mechanism by which AID mediates 2′-OMe in the switch region of Iα RNAs. Because both AID and FBL regulate Iα 2′-OMe and R-loop stability, we investigated whether an interaction occurs between these proteins during CSR. We ectopically expressed FBL and AID in 293 T cells and performed co-immunoprecipitation (co-IP) assays. We found that FBL stably associated with AID (Fig. 4A and Additional file 1: Fig. S4A). To examine the endogenous interaction, we stimulated CH12F3 cells with CIT. Again, we found that FBL interacted endogenously with AID (Fig. 4B and Additional file 1: Fig. S4B). Moreover, the endogenous interaction between FBL and AID was further validated in splenic B cells undergoing CSR (Fig. 4C). Typically, FBL associates with additional proteins (NOP56 and NOP58) in the cell to catalyze 2′-OMe of rRNA. To determine if these proteins were associating with AID, we ectopically expressed NOP56, NOP58, and AID in 293 T cells and performed co-IP assays. Our results suggest that AID does not associate with NOP56 or NOP58 (Additional file 1: Fig. S4C).

To determine the critical domains required for the FBL-AID interaction, we generated various deletion constructs and conducted co-IP assays. FBL is a 321-residue polypeptide with an N-terminal RNA-binding GAR domain and a C-terminal methyltransferase domain. We deleted each domain and found that the RNA-binding GAR domain (FBL-D2) but not the methyltransferase domain (FBL-D1), mediated the interaction with AID (Fig. 4D). AID is a 198-residue polypeptide with a well-folded N-terminal cytosine deaminase domain and a flexible C-terminal tail. Based on previous reports that the C-terminal tail of AID is required for CSR and our observations suggesting that FBL influences CSR, we deleted the C-terminal domain of AID (AIDΔC) [45, 46, 55, 56]. Interestingly, we found that deletion of the C-terminal tail of AID abolished its interaction with FBL (Fig. 4E), suggesting that this short C-terminal domain plays a crucial role for the interaction with FBL during CSR. Although FBL and AID form a complex during CSR, we were unable to detect direct binding between purified recombinant FBL and AID proteins, suggesting that additional components may be required to facilitate the interaction. Because the interaction domain on FBL is an RNA-binding motif, we investigated whether RNA mediated the interaction. We performed co-IP experiments in 293 T cells and treated the FBL-associated materials with RNase A. Interestingly, we found that AID was completely dissociated from FBL following RNase A treatment (Fig. 4F), indicating FBL and AID interaction is dependent on RNA.

To examine the requirement of AID–FBL interaction in B cell function, activated CH12F3 cells were depleted of either FBL or AID by siRNAs transfection (Figs. 1D and S2A). The cells were co-transfected with either wild-type or deletion constructs for each protein (Additional file 1: Figs. S4D-E). We observed that CSR and R-loop levels were rescued to control levels in activated CH12F3 cells transfected with wild-type AID/FBL constructs but not in AIDΔC or FBL-D2 transfection cells (Figs. 4G-H). Likewise, 2′-OMe levels as deciphered by increased RTLP signal was rescued to the control levels only in the cells transfected with wild type AID/FBL but not in AIDΔC or FBL-D2 transfection cells (Fig. 4I). The complementation experiments suggest that AID-FBL interaction is important during productive CSR.

### aSNORD1C is required for 2′-OMe

**Fig. 5:**
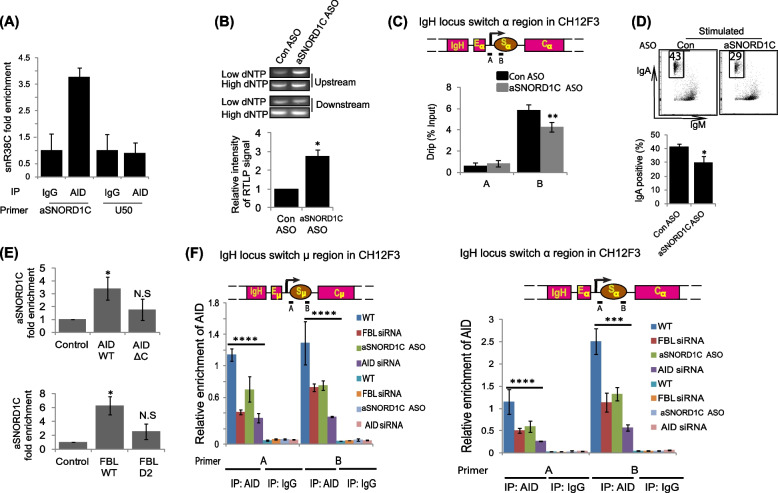
aSNORD1C is required for 2′-OMe. **A** AID associates with aSNORD1C. AID-expressing stable cells were analyzed using CLIP assays followed by qPCR to check for the enrichment of aSNORD1C and U50. Enrichment is represented by snoRNA levels in the immunoprecipitate/relative to snoRNA levels in the input samples. The data are represented as mean of two biological replicates and error bars represent the values of the two replicates. IP: antibodies used for CLIP, primer: primer used for qPCR. **B** The top panel shows the levels of Um1581 as determined by RTLP, after aSNORD1C knockdown using antisense oligonucleotides (ASO). The bottom panel shows the mean of PCR product signal intensity ± standard deviation of three biological replicates. All experiments were conducted independently at least three times. **C** The top panel depicts the schematic representation of the IgA locus, showing the position of the two primers used for quantification of switch RNA-associated R-loops. The bottom panel shows the levels of DNA-RNA hybrids (DRIP) at the IgA locus after ASO mediated aSNORD1C knockdown in CIT-activated CH12F3 cells. ASOs were electroporated into the cells, and the cells were grown for 24 h before immunoprecipitation was carried with S9.6 antibodies. Pull-down efficiency was calculated using total input DNA as a normalization control. Error bars represent the standard deviation of three independent experiments, **p ≤ 0.01. **D** aSNORD1C is required for optimal CSR. The left panel shows the flow cytometry analysis of IgM and IgA on the surface of stimulated CH12F3 cells after ASO-mediated aSNORD1C knockdown. CH12F3 cells were transfected with either control (Con) ASOs or a mixture of ASOs targeting aSNORD1C, grown for 24 h, stimulated with CIT for 48 h. The percentage of cells switching to IgA is indicated in the boxes within each plot. The right panel shows the mean levels of switching ± standard deviation of three biological replicates. **E** aSNORD1C interacts with wild-type AID and FBL but not with AIDΔC and FBL-D2. CH12F3 cells were transfected with respective siRNAs and co-transfected with expression constructs of wild-type or mutant proteins. The ectopic-protein and RNA complexes were enriched by RNA immunoprecipitation. aSNORD1C enrichment was checked by qPCR represented by snoRNA levels in the immunoprecipitate/relative to snoRNA levels in the input samples. The data are represented as mean ± standard deviation of three biologically independent experiments, *p < 0.05. **F** aSNORD1C and FBL recruit AID to the S region DNA. ChIP analysis of the Sμ and Sα regions. The top panel shows schematic representation of the Igμ and Igα loci and the position of primers (A and B) within the respective switch regions used for PCR amplification. CH12F3 cells were transfected with respective siRNAs for 24 h. The cells were then CIT stimulated and the immunoprecipitates were collected 24 h later using anti-AID antibodies or anti IgG antibodies. The immunoprecipitates were analyzed by qPCR and relative enrichment was calculated using immunoprecipitate/relative to the input samples. The data represents mean ± standard deviation of three biologically independent experiments *p < 0.05. The statistical significance was determined using a two-tailed Student’s *t*-test and error bars represent ± standard error of the mean

Previous studies have shown that FBL interacts with snoRNAs, which target FBL to its 2′-OMe substrates, such as rRNA [38, 39]. Thus, we investigated whether snoRNAs could be involved in mediating the interaction between FBL and AID during 2′-OMe of switch transcripts. Homology-directed searching in the snOPY [57] target prediction database suggested that the Sα Um1581 could act as a substrate for the snoRNA SNORD1C (NCBI reference number: Human NR_004397.2, Mice NR_028569.1) (Fig. S5A). However, sense IgA is complementary SNORD1C in antisense orientation. To check for the presence of antisense SNORD1C (hereafter referred to as aSNORD1C), we carried directional RT-PCR on RNA isolated from HEK293 and CH12F3. We observed that both sense and antisense SNORD1C were expressed by HEK293 and CH12F3 cells (Fig. S5B). In order to investigate the association between IgA and aSNORD1C, we established a stable AID expressing 293 T cell line (Additional file 1: Fig. S5C) and performed crosslinking-immunoprecipitation (CLIP) assays. As suspected, qPCR quantification of the immunoprecipitates revealed 3.7-fold enrichment of aSNORD1C in cells stably expressing FLAG-AID cells compared to parental HEK293 cells. As the negative control, we monitored enrichment of the unrelated snoRNA U50 and we did not observe any difference between wild type and AID-stable cell line (Fig. 5A). These results suggest that AID could be responsible for binding of guide snoRNAs during FBL-mediated 2′-OMe on switch transcripts. To test this hypothesis, we knocked down aSNORD1C using antisense oligonucleotides (ASO) in CSR-stimulated CH12F3 cells (Additional file 1: Fig. S5D). The levels of 2′-Ome on Um1581 were reduced as evidenced by a threefold increase in the RTLP signal with respect to control. Likewise, R-loops at the IgA region were reduced after knockdown of aSNORD1C (Figs. 5B-C). In addition, loss of aSNORD1C significantly reduced CSR to IgA in CSR-stimulated CH12F3 cells (Fig. 5D). SNORD1C is known to guide 2′-O-methylation of G4044 (Gene bank: X00525.1) in mice 28S rRNA (G4362 in human 28S rRNA) [57]. To check if aSNORD1C knockdown can influence G4044 2′-OMe, we performed RTLP analysis of the methylation site in the CH12F3 knockdown cells. Our results suggest that knockdown of aSNORD1C does not influence the 2′-OMe of G4044 (Additional file 1: Fig. S5E).

Next, we screened whether additional snoRNAs within the cells can guide global S regions 2'-OMe, we downloaded sequences of all the snoRNAs from the snOPY [57] database. By blasting the SnoRNA with the S regions of IgA (GenBank: D11468.1) and IgG (GenBank: M12389.2), we identified five candidate base complementarities for each S region which was located in G-rich regions (GG beginning and GG ending) (Additional file 1: Fig. S6A). By blasting the five conserved motifs with the sequences of IgM S region (GenBank: M28469.1) and IgE S region (GenBank: J00477.1), we found that the “GGGUCGGG” target sequence was conserved across multiple S regions (Additional file 1: Fig. S6B-E). Thus, it is likely that snoRNAs with “GGGUCGGG,” such as SNORD23 and SNORA5, and additional snoRNAs can function in the 2′-OMe of immunoglobulin S regions. These in silico experiments would need to be supported by experiments to validate the presence of these modifications and with the current experimental data, we cannot infer if these sites represent true or false modification sites of the Ig loci.

We next performed ectopic expression of wild-type AID/FBL or deletion mutant AIDΔC/FBl-D2 constructs in activated CH12F3 cells and enriched the interacting RNAs by RNA-immunoprecipitation (RIP) assays. We observed a significant reduction in aSNORD1C interaction in AIDΔC and FBl-D2 constructs in comparison to wild-type proteins (Fig. 5E).

The ability of AID to bind aSNORD1C and FBL during CSR and the role of this complex in mediating the 2′-OMe of switch transcripts at the S region DNA which also serve as a deamination target for AID prompted us to investigate the requirement of this interaction in mediating AID targeting to these S regions. We observed that in CIT-stimulated CH12F3 cells, knockdown of aSNORD1C or FBL decreased occupancy of AID at both Sμ and Sα regions (Fig. 5F). In addition, we performed ChIP assay to check the levels of FBL at the IgH locus upon AID knockdown and observed that recruitment of FBL was not impaired upon AID knockdown (Additional file 1: Fig. S6F). These results indicate that aSNORD1C and FBL promote AID recruitment at the S regions. Finally, R-loop abundance in CSR stimulated cells is directly dependent on the transcription of the germline transcripts formed at the IgH loci. We thus checked the levels of these germline transcripts upon knockdown FBL, AID, and aSNORD1C using nuclear run-on assay. Our results suggest that the levels of the transcripts were not significantly changed upon the knockdown suggesting that the observed changes in R-loop abundance, 2′-O-methylation, and CSR are not due to non-specific decreases of germ line transcripts (Additional file 1: Fig. S6G). In addition, we checked the levels of AID and FBL to make sure that the knockdown was specific and not due to any off targeting of the siRNA/ASO. Our results suggest that the oligos are specific in their targeting (Additional file 1: Fig. S6H).

Collectively, our study demonstrates that the GAR domain of FBL associates with C-terminal region of AID via aSNORD1C and that this interaction promotes the recruitment of AID to the IgH locus and enables FBL to methylate switch transcripts for optimal R-loop abundance and CSR (Fig. S7).

### AID does not deaminate 2′-OMe RNA

CSR is accomplished by a genetic rearrangement initiated in response to AID-mediated deamination of dC into dU in the single-stranded DNA strand. The presence of dU in the DNA is sensed by DNA damage repair proteins leading to the breakage of DNA. This model in which DNA acts as a deamination target of AID is known as the “DNA model” and is supported by majority of studies [12, 45, 58]. An alternative “RNA model” assumes that RNA not DNA acts as the deamination target for AID [59–61]. These studies are based on the high degrees of homology between AID and its ortholog APOBEC1-which is an RNA editing enzyme. AID and APOBEC1 belong to a family of proteins involved in cytidine deamination [62, 63]. However, an in vitro model supporting the RNA target has not been reported for AID. Because our data suggest that switch RNAs could be 2′-O-methylated and that AID interacts with 2′-O-methyl transferase FBL, we incubated synthetic chimeric RNAs containing 2′-OMe cytidine with an in vitro purified AID (Additional file 1: Fig S8A). Although AID was successfully able to deaminate cytidine into deoxy-uracil on DNA (Additional file 1: Fig S8B), we did not observe the formation of 2′-O-methylated uracil when the AID substrate was chimeric RNA (Additional file 1: Fig. S8C). These results are in agreement with previous studies suggesting that 2′-O-modifications on the RNA could be responsible for the lack of deamination activity of AID on RNA [64] and further support the hypothesis that AID cannot deaminate 2′-O-methylated RNA/cytidine.

## Discussion

Here, we report that the IgH-associated R-loops are modified with 2′-OMe which in turn stabilizes them and prevents their collapse during productive CSR. We also suggest that R-loop 2′-OMe is catalyzed by FBL and guided by an AID-associated snoRNA aSNORD1C. The association of AID with aSNORD1C and FBL mediates targeting of AID to the IgH locus.

Previous studies of CSR have implicated R-loop functions in both the initiation and the final recombination events of CSR [5, 16]. CSR initiation studies favor a model in which IgH-associated R-loops help target AID to its single-stranded DNA substrate. However, this model cannot explain the deamination of the non-template strand. A recent report suggests that stable G4 structures are formed by the non-template strand and the switch RNA and that these structures contribute to the targeting of AID and looping of the immunoglobulin locus [8, 20]. Although the exact steps of CSR that are influenced by R-loops are actively debated, proponents of both models agree that R-loops promote successful CSR. Nonetheless, R-loops can lead to genomic instability and consequently are rapidly degraded by RNase H-mediated digestion of the RNA component. However, the mechanism that prevents RNase H-mediated digestion and promotes the persistence of IgH-associated R-loops has not been explored. We found that the IgA switch region RNA harbors 2′-OMe (Fig. 1A). Based on previous studies showing that RNase H-mediated degradation of RNA/DNA hybrids can be prevented if the RNA component is modified by 2′-OMe [48, 65], we propose these modifications prevent the degradation of IgH-associated R-loops.

We used oligonucleotide-hybridization-based biochemical methods to purify Iα RNA and employed primer extension to map Um1581 on the IgA switch region. RTLP assays showed that Um1581 modification was present in the RNA within R-loop itself (Figs. 1B, D). We also observed that the IgA RNA U1581 site was resistant to RNAse H-mediated digestion (Fig. 1C) and based on previous reports that 2′-OMe has an inherent ability to resist RNase H digestion [15, 66], we propose that 2′-OMe of IgH-associated R-loops can favor their selective persistence. Although here we have focused on mapping 2′-OMe in the IgA switch region; however, 2′-OMe was also observed in Iμ RNA suggesting that this modification could be present across all immunoglobulin switch loci. Since we observed a dominant band at Um1581, we focused our studies on this locus. Thus based on our analysis at multiple switch regions, we expect that other 2′-OMe sites could potentially regulate R-loop stability at non-IgA switch regions. All Nm modifications across the Ig loci may be collectively engaged to resist RNAse H digestion. In addition, although we focused our studies on Um158, we speculate that our knockdown strategies would be affecting the levels of Nm across the whole switch loci. Nevertheless, future studies deciphering a complete 2′-OMe mapping profile of the IgH-associated R-loops is required for complete understanding of the role of R-loop modification in CSR. In addition, our results do not contradict previous studies in which endogenous switch loci were inverted or replaced with artificial sequences capable of forming R-loops. We hypothesize that Nm modification, being the most abundant RNA modification in the cell along with the snoRNAs themselves which are highly abundant in the cells, may be involved in Nm modifications of the GC-rich regions irrespective of the specific sequence requirements [67]. Thorough studies of these ideas are beyond the scope of the current investigations. Notably, we did not observe a significant enrichment of 2′-OMe in the R-loops in a global context (Fig. S1C). This observation is highly likely due to insufficient enrichment of RNA by the DRIP method which was employed during our study.

 Interestingly, we observed that FBL is required for 2′-OMe of Um1581 and knockdown of FBL reduced 2′-OMe levels at the target loci (Fig. 1E). In contrast, loss of other putative cellular 2′-O-methyltransferases like FTSJ2, FTSJ3, and CMTR2 had no effect on 2′-OMe enrichment of Um1581. Additionally, our data proposes a working model in which FBL associates with AID during global CSR (Figs. 4B-C). Previous studies suggest that FBL itself has low affinity for RNA and associates with additional proteins including NOP56 and NOP58 to form a methylation complex that in turn is directed by a guide snoRNA during 2′-OMe. Likewise, our study suggests that FBL forms a complex with AID using aSNORD1C as the guide to deposit 2′-OMe on Um1581. AID bound aSNORD1C potentially changes the conformation of the FBL RNA-binding domain (GAR domain) or modulates the structure of aSNORD1C in the AID-aSNORD1C complex and thereby helps FBL bind aSNORD1C. In agreement to our hypothesis, we observed that knockdown of AID or aSNORD1C led to reduced levels of 2′-OMe on Um1581 (Figs. 3F and 5B). We next uncovered a physiologically functional interaction between AID and FBL and observed that the association between AID and FBL was abolished upon RNase A treatment (Fig. 4F) suggesting a possible role for RNA in mediating the interaction between AID and FBL. Previous studies involving in vitro purification of AID have also demonstrated the role of regulatory RNAs in modulating the function of AID [9, 29, 56]. However, the identity of these RNAs has not been revealed yet. Here, we identify aSNORD1C as the bridging RNA, forming a direct complex between AID and FBL through their C-terminal and GAR domain, respectively. We used complementation experiments to map the interaction domains of AID and FBL facilitating aSNORD1C interaction (Fig. 5E). In addition to aSNORD1C, it is also likely that other snoRNA/RNAs may mediate the interaction between AID and FBL at other switching loci. Apart from aSNORD1C, our bioinformatic analysis uncovered potential snoRNAs which can function at other switch loci (Fig S6A-E). In our proposed model, FBL interacts with AID via its RNA-binding domain and loss of this domain completely abolishes the interaction (Figs. 4 and 5). The RNA-binding domain-based requirement of this interaction further indirectly supports our hypothesis that the association between AID and FBL is RNA-dependent. Nevertheless, we identified aSNORD1C and AID as essential ribonucleoprotein factors required for 2′-OMe on Um1581, and it is highly likely that FBL uses an evolutionarily conserved pathway during the 2′-OMe deposition on Um1581. The aSNORD1C-AID-FBL complex would be recruited to the switch regions due to base pairing of aSNORD1C with switch RNA. A closer examination of the fibrillarin target site on IgA (Fig. S5A) identifies a novel methylation pattern. Fibrillarin is well known to methylate RNA immediately upstream of a box D (CUGA) or D′ in the guide snoRNA [37, 68]. However, in the present model, we observe a deviation in the fibrillarin action in that a D/D′ is not located immediately upstream of the target methylation site. We suspect the deviation from the canonical box C/D guide RNAs in our study could be possibly due to the involvement of AID in FBL-snoRNA complex as well as due to the lack of NOP56 and NOP58 within this complex. However, additional studies are required to fully decipher this unique target selection.

We next demonstrated a clear and direct relationship between R-loop stability and 2′-OMe. However, FBL knockdown not only decreased R-loops at IgA locus but at IgM locus as well (Figs. 3A-E). The ability of FBL to regulate the abundance of IgM-associated R-loops suggests that it seems reasonable to assume that FBL mediated 2′-OMe of the IgM-associated R-loops could be functional at the IgM locus. However, the experiments reported here did not explore the identity of 2′-OMe on the IgM region. In agreement to our hypothesis, we observed that reduced FBL expression perturbed R-loop levels at both IgM and IgG1 loci in splenic B cells.

Surprisingly, R-loop levels were dependent on AID expression (Figs. 3A-D). In addition, AID-associated with aSNORD1C via its C-terminus and loss of the C-terminus abolishes the association. Although, the role of AID C-terminal tail in regulating CSR function is undisputed, the mechanistic function of the region was not properly deciphered until recently [69]. Recent studies suggest that, AID C terminal deletion constructs lack IgH specific targeting and forms condensates within the nucleolus due to its intrinsic sequence features. In contrast, wild-type AID actively shuttles between nucleus and cytoplasm wherein it interacts with RNAs and the associated ribonuclear proteins that provides a specialized compartmentalization for AID specific targeting [69, 70]. We observed that AID C-terminus associates with aSNORD1C and this association favors recruitment of AID to the S region DNA and that the recruitment was promoted by FBL (Fig. 5). The modification of the switch RNAs by AID-aSNORD1C-FBL complex may be required for facilitating IgH-specific AID targeting and thereby preventing global deamination.

## Conclusions

In conclusion, our results suggest that RNA 2′-OMe stabilizes IgH-associated R-loops to enable productive CSR. These results would shed light on AID-mediated CSR and explain the mechanism of R-loop formation, stability, and turnover.

## Methods

### Cell culture, CSR assay, siRNA, and LNA gapmer transfection

CH12F3 (5 × 10^4^ cells/ml) and naïve splenic B cells (0.5 × 10^6^ cells/ml) were cultured in complete RPMI 1640 containing 100U/ml penicillin and 10% heat-inactivated fetal bovine serum (FBS). CH12F3 cell culture was supplemented with 0.1 M MEM non-essential amino acid, 2 mM L-Glutamine, 50 μM β-mercaptoethanol, and 20 mM HEPES. HEK293T cell line was maintained in standard DMEM media.

CH12F3 cells were transfected with siRNA (Stealth siRNA/Silencer® siRNA pool-Thermo Fisher) using electroporation and allowed to grow for 24 h. The cells were stimulated to IgA using 1 μg/mL anti-CD40, recombinant IL-4 20 ng/mL, and 1 ng/mL TGF-β (referred to as CIT treatment) for the indicated time points. Naïve splenic B cells (C57BL/6) were isolated from the spleen of 10–12-week-old C57BL/6 mice using CD43-negative selection (Dynabeads™ Mouse CD43), RBCs were removed, and the cells were stimulated to undergo CSR to IgG1 using 1 μg/mL anti-CD40, 20 ng/mL recombinant IL-4, and 20 μg/mL LPS (CIL treatment) for 24 h. The next day, electroporation was used to transfect the cells with the respective siRNAs (Stealth siRNA/Silencer® siRNA pool-Thermo Fisher) and allowed to grow for the indicated time points. For the gapmer assay, the cells were transfected with the LNA oligos and stimulated as as described above and the cellular RNA was isolated 24 h post stimulation and quantified by qPCR.

### Flow cytometry

CSR efficiency was monitored by FACS analysis using BD Fortessa (BD Biosciences) and FlowJo software. For CH12F3 cells, CSR efficiency was checked 48 h post stimulation while splenic B cells were analyzed 72 h post stimulation. For FACS analysis of CH12F3, phycoerythrin (PE)-conjugated IgA and FITC-conjugated IgM antibodies were used. Splenic B cells were analyzed using PE-conjugated B220 (B cell marker) and FITC-conjugated IgG1 antibodies. The flow cytometry data represents 2000–5000 live cell events as determined by forward scatter and DAPI gatings. Cellular proliferation post transfection was monitored using CellTrace™ CFSE Cell Proliferation Kit following the manufacturers’ guidelines.

### Protein purification and in vitro assay

Human AID (UniProt ID: Q9GZX7) was cloned into a pFastBac vector (Thermo Fisher) with a GST tag and recombinant baculoviruses were generated (Invitrogen). The baculoviruses were expressed in Sf9 insect cells for 48 h. Cells were collected and washed with ice-cold PBS and lysed in NETN-100 buffer (20 mM Tris–HCl pH8.0, 1 mM EDTA, 100 mM NaCl, 0.5% Nonidet P-40). The soluble lysate was collected and 30 µl slurry of glutathione-sepharose-4B-beads (Thermo Fisher) was added and incubated at 4 °C for 2 h on a shaker. The beads were collected, washed five times with NETN-100 buffer followed by elution of the protein using glutathione. The eluted protein samples were dialyzed with 20 mM Tris (pH 7.5), 10 mM NaCl, 20% glycerol, and 0.1 mMDTT. The molecular weight of the purified GST-AID was ≈ 50 kDa.

The deamination assays involving DNA and RNA was performed by primer elongation and dideoxy-nucleotide termination as described by Ronda Bransteitter [9]. The deamination assay on DNA was perfomed using Sequenase™ Version 2.0 DNA Sequencing Kit (Thermo Fisher). The RNA deamination was performed using avian myeloblastoma virus (AMV) reverse transcriptase. Both ssRNA and RNA–DNA hybrids were analyzed in the deamination assay of RNA. The deamination products were run on 8% polyacrylamide–8 M urea sequencing gel. The sequence of the synthetic oligonucleotides used for these assays are shown in the Additional file 2: Table 1.

### SDS–polyacrylamide gel electrophoresis (PAGE) and immunoprecipitation

Equivalent amounts of proteins as determined by Bradford method were used in all blotting assays. Briefly cells were lysed in NETN-100 buffer (20 mM Tris–HCl pH8.0, 1 mM EDTA, 100 mM NaCl, 0.5% Nonidet P-40) supplemented with cocktail of protease inhibitors. The lysate was collected by centrifugation (15,000 rpm for 15 min) and 30 μl slurry of protein A beads or protein G beads, along with the respective immunoprecipitating antibody, was added into equivalent amounts of the protein and incubated at 4 °C overnight on a rocker shaker. The beads were collected, washed five times with NETN-100 buffer, boiled at 95 °C for 5 min, and finally suspended in SDS sample loading buffer. The samples were then subjected to polyacryl-amide gel electrophoresis and analyzed with indicated antibodies in Figs. 4 and S4.

To determine the association between AID and the cellular FBL, human FLAG-tagged AID (pS-Flag-SBP) and HA-tagged (pCMV-HA-N) FBL were constructed and transfected into HEK293T cells using Lipofectamine 2000. Co-IPs was performed on the cell lysates 24 h after transfection. For domain mapping, an AID C-terminal deletion construct (amino acids 1–172) was constructed in pS-Flag-SBP vector, whereas FBL deletion constructs D1 (GAR domain) and D2 (methyltransferase domain) were constructed in pCMV-HA-N vectors. CH12F3 and splenic B cell were either stimulated or unstimulated to under CSR as discussed and the co-IP was performed with the indicated against AID (Thermofisher Scientific catalog no. ZA001) and FBL (Santa Cruz, catalog no.sc-25397) (Figs. 4 and S4).

### DNA/RNA immunoprecipitation (DRIP) assay

DRIP was performed as described (Ginno et al., 2012) [71] according to the protocol kindly provided by Dr. Yanzhong Yang and Dr. Qais Al-Hadid (City of Hope). Equal numbers of CH12F3 and splenic B cells were transfected and stimulated as described above. The cells were collected at the indicated time points and washed with PBS and lysed gently using 20% SDS in TE buffer followed by Proteinase K digestion at 37 °C overnight. The next day, the genomic DNA was extracted using phenol/chloroform/isoamyl alcohol (PCI). The DNA was spooled gently on a pipette tip, washed five times with 70% ethanol, and dried for 3 min at room temperature. The DNA was diluted in TE buffer and digested with HindIII, XbaI, SspI, EcoRI, and BsrGI in NEB buffer 2.1 (50 mM NaCl, 10 mM Tris–HCl, 10 mM MgCl_2_, 100 μg/ml BSA [pH 7.9]) overnight at 37 °C. The next day, the DNA was digested with RNase A (1 mg/ml) at 37 °C for 1 h. The DNA was extracted again using PCI and 10 μg of the DNA was digested with RNase H (20U) overnight at 37 °C and collected the following day. Both RNase H-treated and untreated samples (4 μg) were immunoprecipitated with 10 µl of S9.6 antibodies (Kerafast) in 10 × binding buffer (140 mM NaCl, 10 mM NaPO4 [pH 7.0] and 0.05% Triton X-100) at 4° C overnight on a rocker shaker. Protein A/G agarose beads were added into the sample to capture antibody-R-loop complexes for 2 h and then the beads were washed five times with 1 × binding buffer. The DNA was extracted using DNA purification kit and analyzed by real-time PCR.

Same procedure was used for total R-loop isolation from activated CH12F3 cells. The enriched R-loops were subjected to DNase I digestion and the RNA was isolated using Quick-RNA Miniprep Kit (Zymo). The RNA was then analyzed for 2′-O-methylation status using RTLP assay as described later in the method section.

Similarly, global 2′-O-methylation levels were determined by isolating total R-loops using the DRIP assay. The DNA was removed by DNase I digestion and the RNA was isolated. The RNA was nucleosides and subjected to LC MS/MS as described later in LC/MS analysis.

### Chromatin isolation by RNA purification (ChIRP)

Iα RNA was purified using modified ChIRP protocol as described by Chu et al. [72]. Roughly one billion CH12F3 cells were unstimulated or stimulated with CIT to under CSR to IgA as described above. RNA specific probes (31 probes for IgA and 23 probes for IgM) were designed using Biosearch Technologies Stellaris FISH Probe Designer available online. The masking level during the probe design was set to maximum (i.e., 5) for highly specific probe selection. Moreover, the hybridization was carried at elevated temperatures (55 °C) for 2 h. The beads were washed (buffer composition: 2 × NaCl and Sodium citrate, 1% SDS) ten times. The cells were collected and the Iα RNA was enriched as described in the protocol. The rRNA was depleted from the enriched RNA using two RiboMinus™ treatments following manufacturers’ instructions.

### LC/MS analysis

Switch RNAs were isolated from untreated and CIT-treated CH12F3 cells using the modified ChIRP protocol with biotin-labeled probes as described above. The RNA was then subjected to LC/MS analysis as described by Chen et al. [73]. Briefly, the extracted RNA was digested into nucleosides using phosphodiesterase (PDE), alkaline phosphatase (AP), and benzonase (Sigma) at 37 °C for 4 h. The nucleosides were separated using HPLC followed by analysis on Agilent 6520B QTOF mass spectrometer with analytic column (Phenomenex, size 150 × 2 × 3 micro) at a maximum pressure of 300 bars. Nucleosides were separated using two buffer combinations: solution A (0.1% formic acid in water) and B (acetonitrile). Mass spectra were recorded in positive ion mode with collision energy of 20 arbitrary units. The deamination experiment involving AID and 2'O-Me RNA was performed as described above “Protein purification and in-vitro assay”. The RNA was then digested and subjected to LC–MS/MS analysis.

### Primer extension and reverse transcription at low deoxyribonucleoside triphosphate concentrations (RTLP)

CH12F3 cells were either unstimulated or stimulated with CIT, and the I Iα/Iμ RNA was enriched using ChIRP protocolas described above. The RNA was subjected to primer extension at either low dNTP: 0.004 mM or high dNTP: 0.5 mM concentrations [74] with some modifications. The RNA was heat denatured at 75 °C for 10 min and immediately chilled it on ice. We then carried reverse transcription at elevated temperatures (68 °C) using AMV RT (Promega cat. no. M9004). The presence of 2′-O-methylated bases leads to a strong bump in reverse transcriptase, and a dominant band of cDNA is formed. For truncated cDNA library preparation, gene-specific primers were used to perform reverse transcription, and the 5′ ends of cDNA libraries were captured using template-switching oligonucleotides (IDT). The cDNA libraries were cloned into pMD20 vectors (TakaRa) and 230 clones were Sanger sequenced. To map the position of 2′-O-methylated bases, radiolabeled gene-specific primers were used in the reverse transcription reaction. The cDNA was run on 8% polyacrylamide-8 M urea sequencing gels. In order to determine the position of 2′-O-methylated bases, a dideoxy sequencing ladder was prepared using Sequenase™ Version 2.0 DNA Sequencing Kit (Thermo Fisher), according to the manufacturers’ guidelines and electrophoresed alongside the primer extension products.

RTLP assay was carried as described by Dong et al. [40]. Briefly, CH12F3 cells were transfected with the respective siRNAs and stimulated using CIT as described, and total cellular RNA was isolated using TRIzol® Reagent. Gene-specific primers (upstream and downstream) for the 2′-O-methylateduridine were designed and used to convert the RNA into cDNA at either high (0.5 mM) or low dNTP (0.004 mM) concentration. The cDNA was PCR amplified. Due to the repetitive nature of the switch region, multiple primers were designed and checked for the specificity and efficiency of PCR. The signal intensities were calculated using ImageJ software.

### Directional RT-PCR and real-time PCR

Total RNA was isolated from the cells using TRIzol® Reagent and the RNA concentration was checked by a NanoDrop 1000 spectrophotometer. The RNA was treated with DNase I to remove any contaminating DNA. Roughly 100–1000 ng RNA was used to perform reverse transcription using the SuperScript™ III First-Strand Synthesis System (Thermo Fisher). For directional RT-PCR forward (antisense SNORD1C specific), reverse primer (sense SNORD1C specific) or no primer (NT) was used in the reverse transcription reaction. Both sense and antisense primers were mixed and used together during the PCR. Real-time PCR was performed on the cDNA using iTaq™ Universal SYBR® Green Supermix (Bio-Rad) on BioRad CFX96 machine. Three independent experiments were performed in triplicate. The primers used in qPCR are additional reagents used in other assays are summarized in Additional file 2: Table 1.

### Cross-linking and immunoprecipitation (CLIP) and RNA immunoprecipitation (RIP)

HEK293T cells were obtained in FLAG-tagged AID in pIRES2-EGFP as described by Kim et al. [75]. The stable cells along with the parental HEK293T cells were grown in culture media supplemented with 6SG (Sigma). RNA UV cross-linking and immunoprecipitation was performed as described by Hefner et al. [76]. The final eluted RNA was purified using RNA Clean & Concentrator (Zymo Research) and subjected to qPCR as discussed.

RIP assay was performed as described above with some modifications. CH12F3 cells were transected with FLAG-tagged AID, AIDΔC, HA-FBL, or HA-FBL-D2 expression constructs and the cells were CIT stimulated for 24 h and analyzed for the associated RNA as described above.

### Chromatin immunoprecipitation (ChIP)

ChIP assays were preformed according to the protocol described by Upstate (Billerica, MA). CH12F3 cells were transfected with respective siRNAs and grown for 24 h. Next day, cells were CIT stimulated for 24 h followed by fixing of cells in 10% formaldehyde for 10 min, quenching the formaldehyde using glycine. The cells were then sonicated to attain a DNA size 300 to 600 bp. The chromatin fractions were collected using with either anti-AID antibodies or anti-IgG antibodies. Protein A/G beads were used to collect the immunoprecipitates, washed, and eluted. The crosslink’s were reversed and the DNA was purified using QIAquick PCR purification kit.

### Polysome analysis

Polyribosome analysis was carried out as described [77]. CH12F3 cells were transfected with respective siRNAs and stimulated with CIT for 48 h. Then cycloheximide (CHX, 100 μg/ml) was added into the media, and the cells were grown for additional 15 min in 37 °C incubator. The cells were collected by centrifugation and washed with cold PBS two times and all the downstream steps were carried at 4 °C. A day before the cell collection, 12 ml 7% to 47% sucrose gradients were prepared in 50 mM NH_4_Cl, 50 mM Tris–Acetate (pH 7.0), and 12 mM MgCl_2_ in thin wall polypropylene tubes and stored at 4 °C overnight. The cells were suspended in 1 ml lysis buffer containing 1% Triton X-100 (v/v), 3 µl SUPERase In™, 1 µl of CHX (100 μg/ml), and 2 mM DTT. Cell lysis was enhanced by passing the suspension were through a 27-gauge syringe needle, followed by incubation on ice for 10 min. The cell lysate was loaded on to the top of the sucrose gradient in a SW 40 Ti Rotor (Beckman) and centrifuged at 40,000 rpm for 5 h. The tubes were then clamped on a stand, the bottom was pierced using a 21-gauge needle, fractions were collected in a 96-well plate, and the absorbance was read at 260 nm in a NanoDrop 1000 spectrophotometer. The absorbance reading was taken from every third well and the values were plotted.

### Statistical analysis

The statistical significance of pairwise differences was determined using a two-tailed Student’s *t*-test and *P*-values less than 0.05 were considered significant. The data represents three independent experiments and error bars represent the standard deviation (SD) of the mean. Statistical significance was estimated using paired two-tailed Student’s *t-*tests. The statistical details of the figures can be found in the figure legends.

### Supplementary Information

Additional file 1: Figures S1-S8. Fig. S1. IgM contains 2′-OMe and 2′-OMe is not a universal feature of R loops. (A) 2′-O-methylation is present in IgM RNA. The diagram shows LC–MS/MS analysis of enriched IgM RNA from CH12F3 cells. The cells were stimulated with *CIT,* and the RNA was enriched using the ChIRP protocol. The extracted ion chromatogram (EIC) of *m/z* 113.03 (uridine) was derived from the MS/MS scan of 2′-O-methyluridine (Um) at *m/z* 259.09 (test: blue color). The chemical structure and the mass spectral properties of chemically synthesized Um (standard: green color) eluted at 1 min are shown and overlapped with the test sample. (B) Left: Schematic representation of Primer extension performed on an enriched IgA RNA from stimulated cells. Right: Primary sequence data of a clone, the position of Um1581, the primer used for RT and the full-length cDNA product are labeled. Reverse transcription of the IgA RNA stopped at the G residue marked with a stop sign, due to 2′-OMe of the upstream U residue. The TSO primer then hybridized to the truncated cDNA end which was then cloned into a TA cloning vector and sequenced. (C) 2′-O-methylation is not a universal feature of R-loops. Total cellular R-loops were isolated from stimulated CH12F3 cells using DRIP. The DNA within the R-loops was digested and the RNA was purified. The purified RNA was digested and subjected to LC MS/MS. The extracted ion chromatogram (EIC) of *m/z* 113.03 (uridine) was derived from the MS/MS scan of Um at *m/z* 259.09 (test: blue color). Uridine spectral peaks *m/z* 259.09 were present in the RNA obtained from stimulated CH12F3 cells but the spectral peaks *m/z* 259.09 corresponding to Um were absent in the RNA. The chemical structure and the mass spectral properties of chemically synthesized Um (standard: green color) eluted at 1 min are shown and overlapped with the test sample. (D) siRNA mediated knockdown of 2′-O-methyltransferases. Top panel: Total cellular RNA from CH12F3 cells was assayed for the levels of FBL, FTSJ2, FTSJ3 and CMTR1 via qPCR after gene-specific siRNA-mediated knockdown. A scrambled siRNA sequence was used in the control (Con) samples. The mean ± standard deviation of three experiments is shown. Right panel: Protein expression analysis after siRNA-mediated knockdown of methyltransferases using western blot. To confirm knockdown efficiency, stimulated cells were transfected with the respective siRNAs or a control (Con) siRNA and grown for 24 h. The cells were then stimulated with CIL and allowed to grow for 48 h before protein levels were measured using Western blot analysis. The Statistical significance was determined using a two-tailed Student’s t-test and error bars represent ± standard error of the mean. (E) FBL expression analysis in splenic B cells after siRNA-mediated knockdown of FBL. Splenic B cells were stimulated with CIT and allowed to grow for 24 h. The next day, FBL siRNA electroporation was done and cells were allowed to grow for the 24 h and FBL levels were measured using Western blot analysis. Right panel: bar graph shows quantification of FBL protein from three replicates. The Statistical significance was determined using a two-tailed Student’s t-test and error bars represent ± standard error of the mean. Fig. S2. Specificity of siRNA mediated FBL knockdown. (A) Knockdown of AID upon siRNA Transfection (B) CSR efficiency in CH12F3 cells after siRNA mediated AID knockdown. Two additional experiments gave similar results. The cells were transfected with AID and then stimulated with CIT for 24 h and the surface expression of IgM and IgA was measured using flow cytometry. The right panel shows the mean levels of class switching to IgA in CH12F3 cells after AID knockdown from three independent experiments,**:*p* ≤ 0.01. (C) Overlay histogram of carboxyfluorescein succinimidyl ester (CFSE) intensity (i.e. proliferation) of CH12F3 cells transfected with FBL siRNAs or control (Con) siRNA, grown for 24/48/72 h post transfection. (D) Effect of siRNA-mediated FBL knockdown on cellular translation. CH12F3 cells were transfected using FBL siRNA or control (Con) siRNA, grown for 24 h and stimulated with CIT for 48 h. The cell lysate was fractionated in a 7–47% sucrose gradient. The fractions were collected, and absorbance was measured at 260 nm. Error bars are representing standard deviation of the three independent experiments. The Statistical significance was determined using a two-tailed Student’s t-test. Fig. S3. The figure shows the expression of DNA-RNA hybrids (DRIP) in stimulated CH12F3 cells at the IgA locus 24 h after FBL and AID knockdown. One set of samples was treated with RNase H prior to immunoprecipitation using S9.6 antibodies. Pull down efficiency was calculated using total input DNA as a normalization control. Error bars represent standard deviation of three independent experiments. The Statistical significance was determined using a two-tailed Student’s t-test. Fig. S4. FBL associates with AID during CSR. (A) In vitro FBL and AID interaction. We transfected FLAG-tagged AID and HA-tagged FBL into HEK293T using Lipofectamine-2000 and the reversible co-IP was performed on the cell lysates 24 h after transfection. (B) Reversible co-IP of endogenous AID and FBL in activated CH12F3 cells. CH12F3 cells were stimulated with CIT for 48 h, and the cell lysate was analyzed by western blot. (C) *In-vitro* AID association with NOP56 and NOP58. FLAG-tagged AID and HA-tagged NOP56, NOP58 and FBL were transfected into HEK293T cells using Lipofectamine 2000 and reversible co-immunoprecipitation (co-IP) was performed on the cell lysates 24 h after Transfection. (D) Ectopic expression of WT and D2 mutant FBL in CH12F3 cells. The plasmids constructs were transfected into CH12F3 cells using electroporation and the protein expression was checked by western blotting. (E) Ectopic expression of WT and ΔC mutant AID in CH12F3 cells. The plasmids constructs were transfected into CH12F3 cells using electroporation and the protein expression was checked by western blotting. Fig. S5. SNORD1C is required for AID and FBL mediated 2′-O-methylation. (A) Schematic representation of the IgA RNA and aSNORD1C showing the positions of complementarity. The encompassing Um1581 is highlighted by an asterisk. (B) Directional RT-PCR in HEK293T and CH12F3 to check the presence of sense and antisense SNORD1C. Sense (Sen), antisense (Ant) or no primer (NT) was used to reverse transcribe the RNA and the resulting cDNA was amplified using both primers together. (C) Generation of AID stable cell line in HEK293T. The cDNA sequence of human AID was cloned in pIRES2-EGFP vector, transfected into HEK293T cells and the stable cells were selected using *G418*. The recombinant protein was detected by Western blot using anti-Flag antibodies. Mock: parental HEK293T cells, AID: clone with a positive EGFP signal, Western blot with actin antibodies served as a loading control. (D) To confirm the knockdown efficiency of ASOs, stimulated CH12F3 cells were transfected with either SNORD1C ASO or a control (Con) ASO and grown for 24 h. The cells were then stimulated with CIT and allowed to grow for 48 h, and the levels of FBL were measured using qPCR. The change in gene expression of the target genes was quantified with respect to snoRNA U16. Error bars are representing standard deviation of the three independent experiments. (E) aSNORD1C knockdown does not affect 28S rRNA methylation at G4044. CH12F3 cells were transfected with ASO’s against aSNORD1C and the levels of 28S rRNA methylation at G4044 were determined using RTLP. The Statistical significance was determined using a two-tailed Student’s t-test and error bars represent ± standard error of the mean. Fig. S6. snoRNAs can guide global S region. (A) List of IgA and IgG S region complementary snoRNAs. (B-E) snoRNA with “GGGUCGGG” sequence including SNORD23 and SNORA5 share homology across IgA, IgG, IgM and IgE S regions. (F) Knockdown of AID does not impair FBL recruitment. The top panel shows schematic representation of the Igμ and Igα loci and the position of primers (A and B) within the respective switch regions used for PCR amplification. CH12F3 cells were transfected with respective siRNAs for 24 h. The cells were then CIT stimulated and the immunoprecipitates were collected 24 h later ChIP assay was done using anti-FBL antibodies or anti IgG antibodies. The immunoprecipitates were analyzed by qPCR and relative enrichment was calculated using immumoprecipitate/relative to the input samples. The data represents mean ± standard deviation of three biologically independent experiments *:*p* < 0.05. (G) Knockdown of FBL, AID and aSNORD1C does not affect germline transcription. CH12F3 cells were transfected with respective siRNAs and stimulated with CIT for 48 h and the levels of IgA transcription was determined using nuclear run-on assays. (H) Knockdown of AID, FBL and aSNORD1C is specific. FBL, AID and aSNORD1C was knocked down using the respective siRNAs and the levels of AID and FBL was determined in each case using western blot. The Statistical significance was determined using a two-tailed Student’s t-test and error bars represent ± standard error of the mean. Fig. S7. FBL- aSNORD1C promote AID recruitment at IgH locus and the complex (AID-aSNORD1C-FBL) deposits 2'-O-Me on the R-loops thereby promoting persistent R-loop formation. Suggested model depicting the possible mechanism involved in the formation of modified R-loop during CSR. Following the appearance of R-loops at the IgH locus, FBL bound AID recruitment is promoted at the R loop by aSNORD1C. FBL in association with its interacting partners (AID and aSNORD1C) deposits 2′-OMe marks on the RNA. The 2′-O-methylated R-loops now resist RNase H mediated digestion. This phenomenon ensures selective persistence of IgH-associated R-loops for an optimal CSR. Fig. S8. AID does not deaminate 2′-O-methylated RNA. (A) Recombinant human AID was purified from Sf9 insect cells, run on a polyacrylamide gel, and stained with Coomassie blue. A size marker and the GST tag are depicted. (B) The top panel shows schematic representation of AID mediated deamination of dC to dU on a ssDNA. The ssDNA containing dC is incubated with the purified AID. T7 sequenase is then used to polymerize a complimentary DNA strand on the synthetic DNA in presence of three dNTP and either in the presence of ddA or ddG. When the template contains dC, T7 polymerization of that template in presence of ddG leads to chain termination and a strong DNA band is formed, however; when AID converts dC to dU, T7 sequenase can read through the dU and a full-length DNA band is formed. The bottom panel shows the results of AID mediated deamination and the sequence of the DNA used in the assay is shown on the right side of the gel. The position of primer used for chain termination reaction and the conversion of dC to dU is shown. (C) The top panel shows schematic representation of AID mediated deamination of Cm to Um on an RNA. Synthetic ssRNA containing Cm or ssRNA-DNA duplex is used as a substrate for AID mediated deamination as described for DNA in Fig. S7B. The RNA is then reverse transcribed into cDNA using AMV RT enzyme. If the Cm on the template RNA was unaltered upon AID treatment, RT reaction at the Cm will be terminated in presence of ddG leading to the formation of an incomplete cDNA band. However, if AID converts Cm to Um, the RT enzyme will read through the now formed Um and a full length cDNA will be formed in the ddG lane. The bottom panel shows the results of deamination obtained with AID mediated RNA deamination. The sequence of the RNA used in the assay is shown on the right side of the gel.

Additional file 2: Table S1. Oligonucleotides used in this study [78–81].

Additional file 3: Raw data.

## Data Availability

All data generated or analyzed during this study are included in this published article and its supplementary information files (Additional Files 1, 2 and 3).

## Abbreviations

CSR: Class switching recombination
AID: Activation-induced cytidine deaminase
2'-OMe: 2'-O-methylation
CIT: Anti-CD40, IL-4 and TGF-β
CIL: Anti-CD40, recombinant IL-4 and LPS
ASO: Antisense oligonucleotides
