# Supplementary material for: RNA 2'-O-methylation promotes persistent R-loop formation and AID-mediated IgH class switch recombination
Source: BMC Biol. 2024 Jul 8;22:151. doi: 10.1186/s12915-024-01947-5 (PMC11232215; doi:10.1186/s12915-024-01947-5)
Supplement: Supplementary file 1 — Additional file 1: Figures S1-S8. Fig. S1. IgM contains 2′-OMe and 2′-OMe is not a universal feature of R loops. (A) 2′-O-methylation is present in IgM RNA. The diagram shows LC–MS/MS analysis of enriched IgM RNA from CH12F3 cells. The cells were stimulated with CIT, and the RNA was enriched using the ChIRP protocol. The extracted ion chromatogram (EIC) of m/z 113.03 (uridine) was derived from the MS/MS scan of 2′-O-methyluridine (Um) at m/z 259.09 (test: blue color). The chemical structure and the mass spectral properties of chemically synthesized Um (standard: green color) eluted at 1 min are shown and overlapped with the test sample. (B) Left: Schematic representation of Primer extension performed on an enriched IgA RNA from stimulated cells. Right: Primary sequence data of a clone, the position of Um1581, the primer used for RT and the full-length cDNA product are labeled. Reverse transcription of the IgA RNA stopped at the G residue marked with a stop sign, due to 2′-OMe of the upstream U residue. The TSO primer then hybridized to the truncated cDNA end which was then cloned into a TA cloning vector and sequenced. (C) 2′-O-methylation is not a universal feature of R-loops. Total cellular R-loops were isolated from stimulated CH12F3 cells using DRIP. The DNA within the R-loops was digested and the RNA was purified. The purified RNA was digested and subjected to LC MS/MS. The extracted ion chromatogram (EIC) of m/z 113.03 (uridine) was derived from the MS/MS scan of Um at m/z 259.09 (test: blue color). Uridine spectral peaks m/z 259.09 were present in the RNA obtained from stimulated CH12F3 cells but the spectral peaks m/z 259.09 corresponding to Um were absent in the RNA. The chemical structure and the mass spectral properties of chemically synthesized Um (standard: green color) eluted at 1 min are shown and overlapped with the test sample. (D) siRNA mediated knockdown of 2′-O-methyltransferases. Top panel: Total cellular RNA from CH12F3 cells was assayed for th [file 12915_2024_1947_MOESM1_ESM.pdf]

Fig. S1

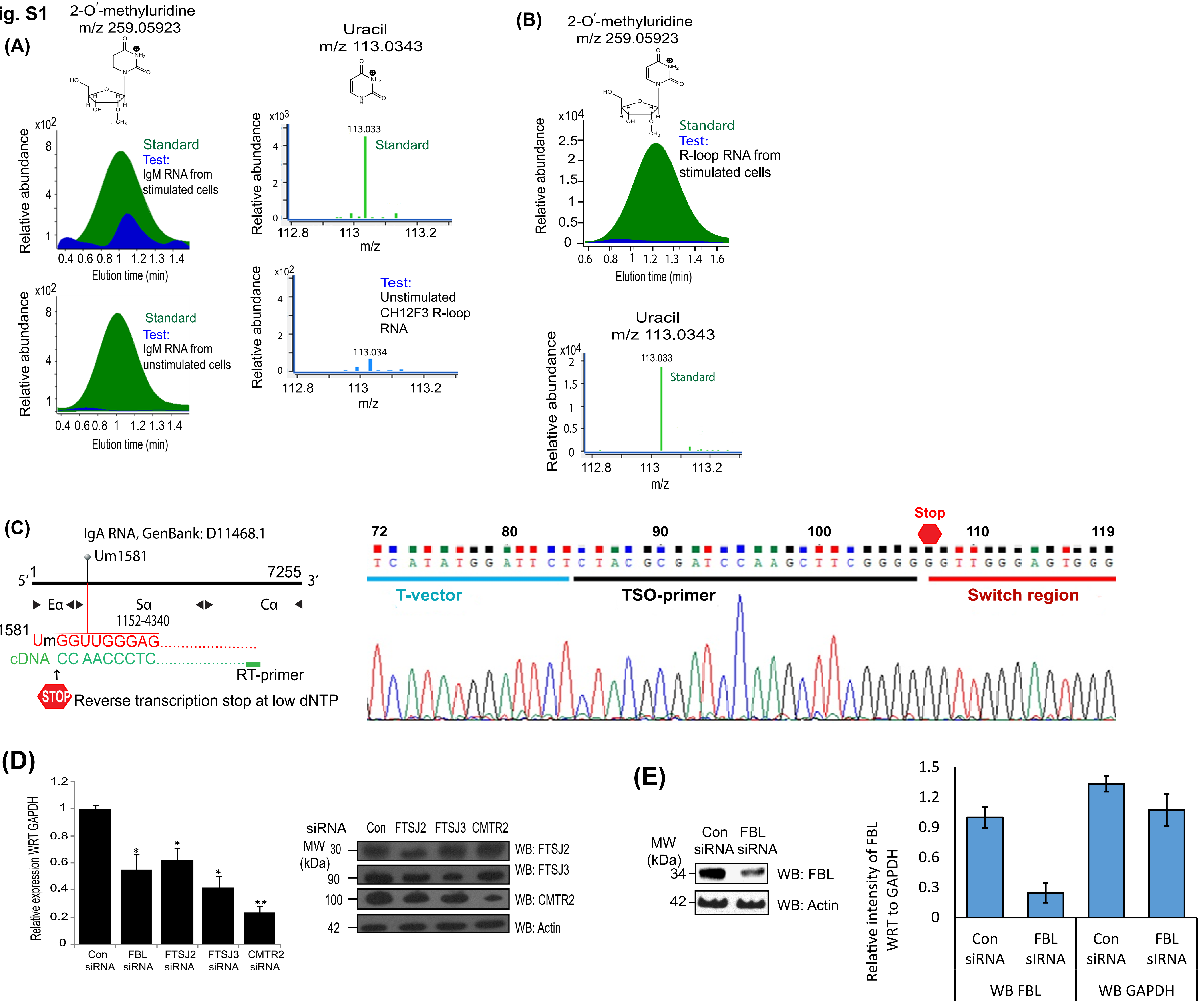

(A)
2-O'-methyluridine
m/z 259.05923
Relative abundance
x10^2
Standard
Test:
IgM RNA from stimulated cells
Elution time (min)
Standard
Test:
IgM RNA from unstimulated cells
Uracil
m/z 113.0343
x10^3
113.033
Standard
m/z
x10^2
Test:
Unstimulated CH12F3 R-loop RNA
113.034
(B)
2-O'-methyluridine
m/z 259.05923
x10^4
Standard
Test:
R-loop RNA from stimulated cells
Elution time (min)
Uracil
m/z 113.0343
x10^4
113.033
Standard
m/z
(C)
IgA RNA, GenBank: D11468.1
Um1581
5'
1
7255
3'
Eα
Sα
1152-4340
Cα
1581
UmGGUUGGGAG.......................
cDNA CC AACCCTC.....................
RT-primer
STOP
Reverse transcription stop at low dNTP
Stop
72
80
90
100
110
119
T-vector
TSO-primer
Switch region
(D)
Relative expression WRT GAPDH
Con siRNA
FBL siRNA
FTSJ2 siRNA
FTSJ3 siRNA
CMTR2 siRNA
siRNA
Con
FTSJ2
FTSJ3
CMTR2
MW (kDa)
WB: FTSJ2
WB: FTSJ3
WB: CMTR2
WB: Actin
(E)
MW (kDa)
Con siRNA
FBL siRNA
WB: FBL
WB: Actin
Relative intensity of FBL WRT to GAPDH
WB FBL
WB GAPDH

Fig. S2

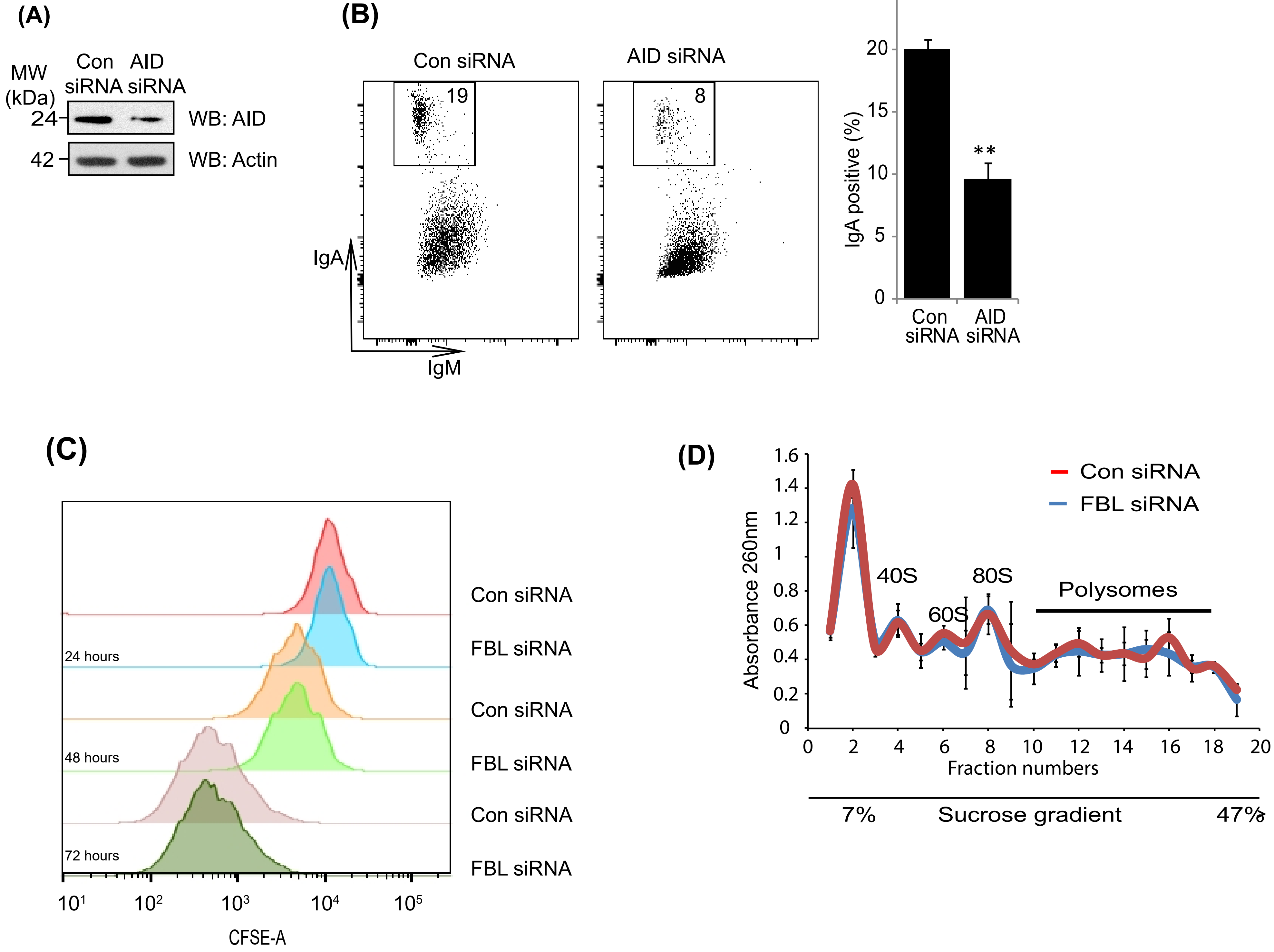

(A)
MW (kDa)
Con siRNA
AID siRNA
24
42
WB: AID
WB: Actin
(B)
Con siRNA
AID siRNA
19
8
IgA
IgM
IgA positive (%)
25
20
15
10
5
0
**
Con siRNA
AID siRNA
(C)
Con siRNA
FBL siRNA
24 hours
Con siRNA
FBL siRNA
48 hours
Con siRNA
FBL siRNA
72 hours
CFSE-A
(D)
Con siRNA
FBL siRNA
Absorbance 260nm
40S
60S
80S
Polysomes
Fraction numbers
7%
Sucrose gradient
47%

Fig. S3

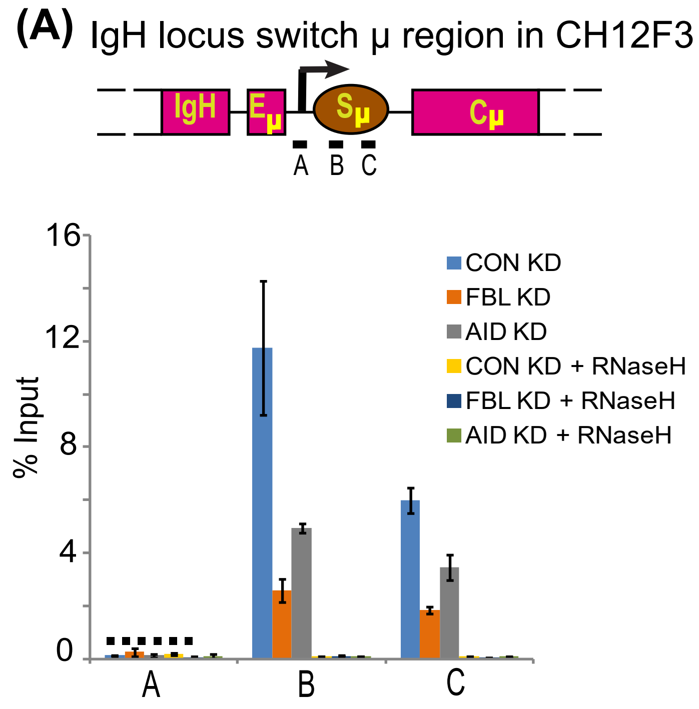

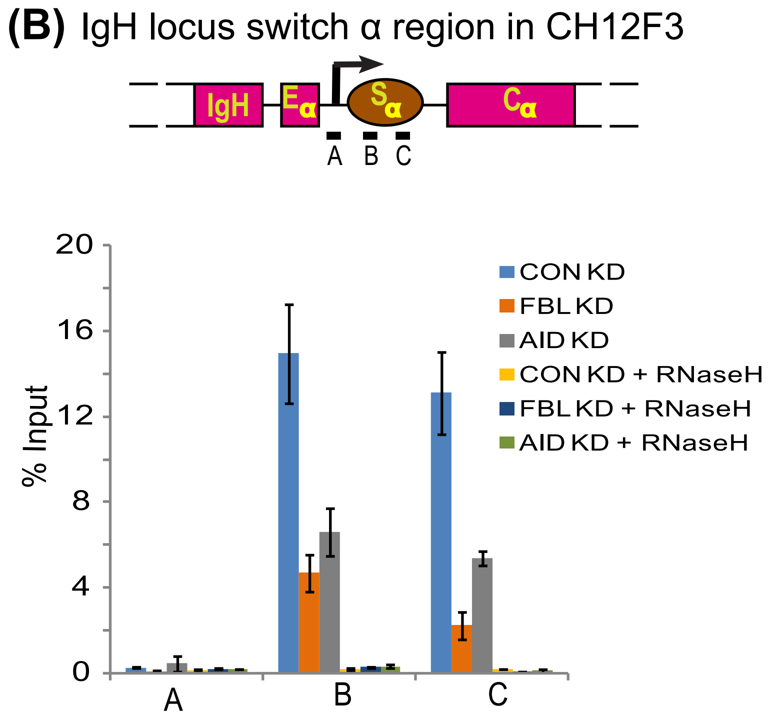

R-loop 24 hours later delete this at end

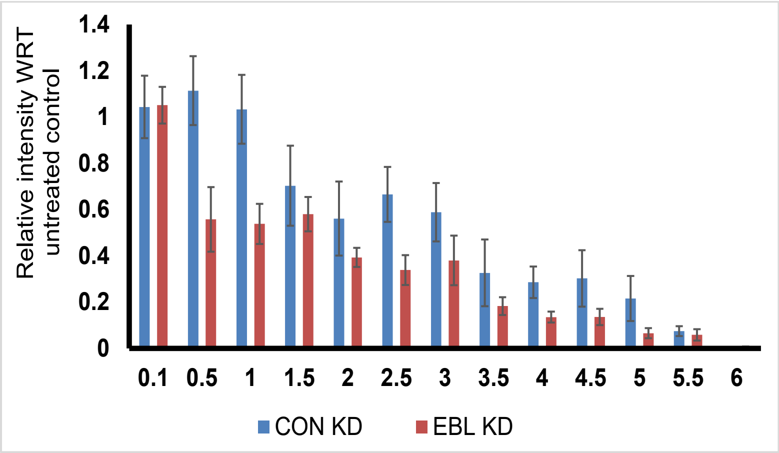

Fig. S4

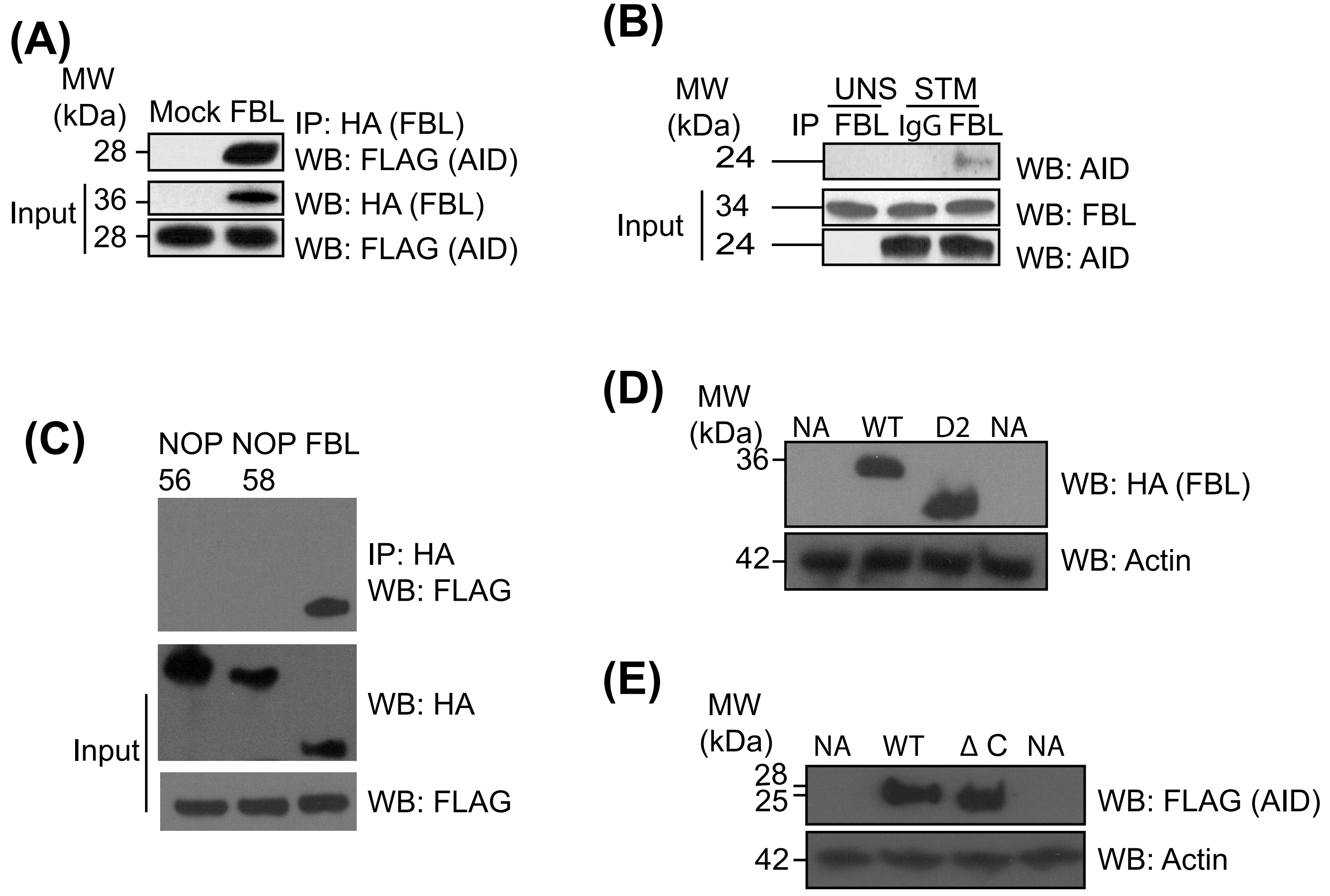

(A)
MW (kDa)
Mock FBL
IP: HA (FBL)
28
WB: FLAG (AID)
36
WB: HA (FBL)
28
WB: FLAG (AID)
Input
(B)
MW (kDa)
UNS STM
IP FBL IgG FBL
24
WB: AID
34
WB: FBL
24
WB: AID
Input
(C)
NOP 56 NOP 58 FBL
IP: HA
WB: FLAG
WB: HA
WB: FLAG
Input
(D)
MW (kDa)
NA WT D2 NA
36
WB: HA (FBL)
42
WB: Actin
(E)
MW (kDa)
NA WT Δ C NA
28
25
WB: FLAG (AID)
42
WB: Actin

Fig. S5

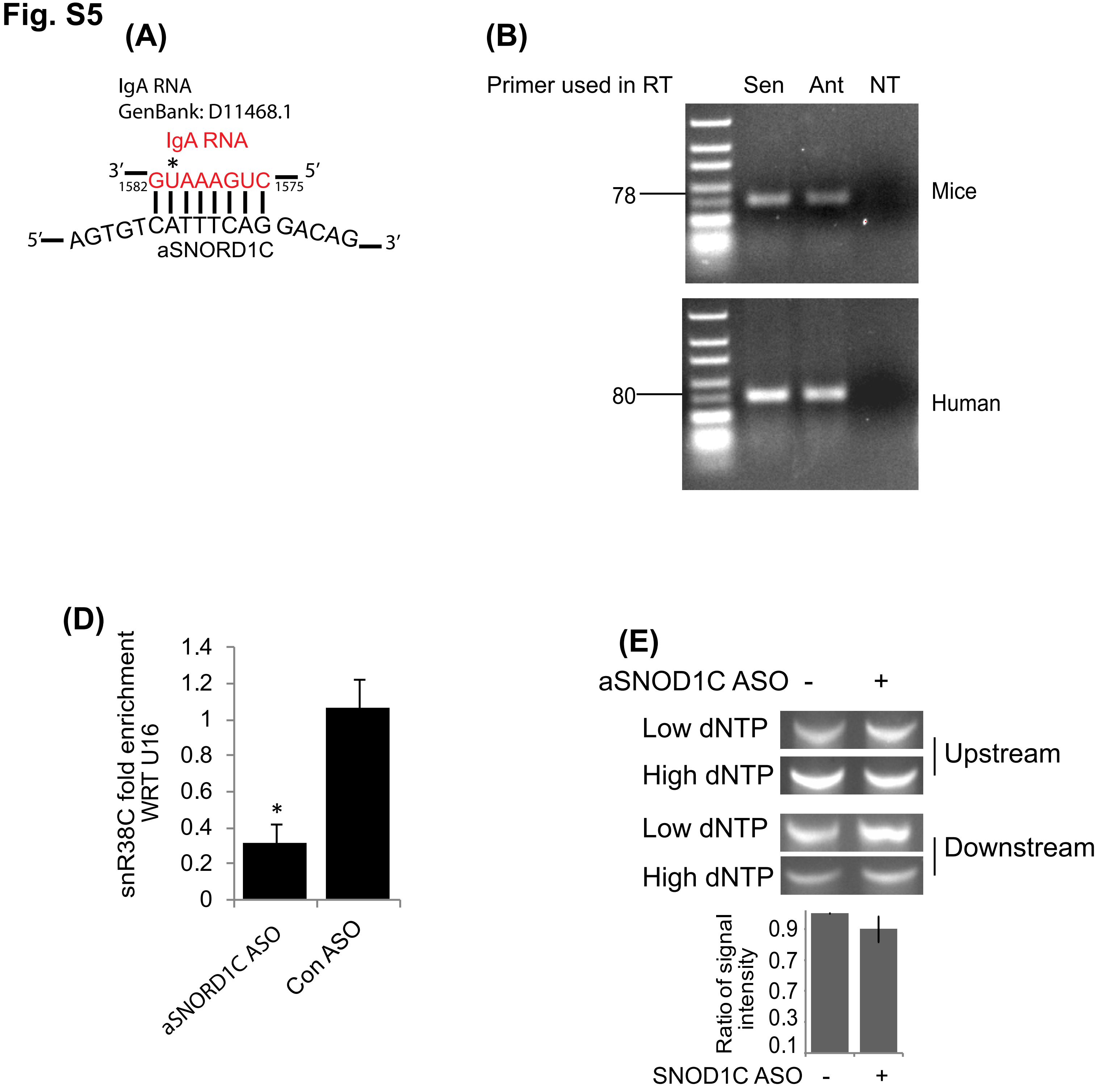
(A)
IgA RNA
GenBank: D11468.1
IgA RNA
3′ 1582 GUAAAGUC 1575 5′
5′ AGTGTCATTTCAGGACAG 3′
aSNORD1C
(B)
Primer used in RT
Sen
Ant
NT
78
Mice
80
Human
(C)
MW (kDa)
Mock
AID
31
WB: FLAG (AID)
42
WB: Actin
(D)
snR38C fold enrichment WRT U16
1.4
1.2
1
0.8
0.6
0.4
0.2
0
aSNORD1C ASO
Con ASO
(E)
aSNOD1C ASO - +
Low dNTP
High dNTP
Upstream
Low dNTP
High dNTP
Downstream
Ratio of signal intensity
0.9
0.7
0.7
0.3
0.1
SNOD1C ASO - +

Fig. S6 **(A)**

IgA S-region		IgG S-region	
Hybridization sequence	snoRNA	Hybridization sequence	snoRNA
CGGGUCGGGU	SNORD23: 300746	AGGGCUGGGG	SNORA5: 300738
GGGUCAGGUG	SNORA68: 300413		SNORA5: 300928
GUCGGACGG	SNORA58: 300905	GGGUCAGGU	SNORA68: 300413
	SNORA94: 300739	GGCAGGGACU	SNORA5: 300908
GGACCGGAU	SNORA43: 300020	GGACCGGGAA	SNORA17: 300853
AGGGUUGG	SNORA32: 300319	AGGGUUGG	SNORA32: 300319

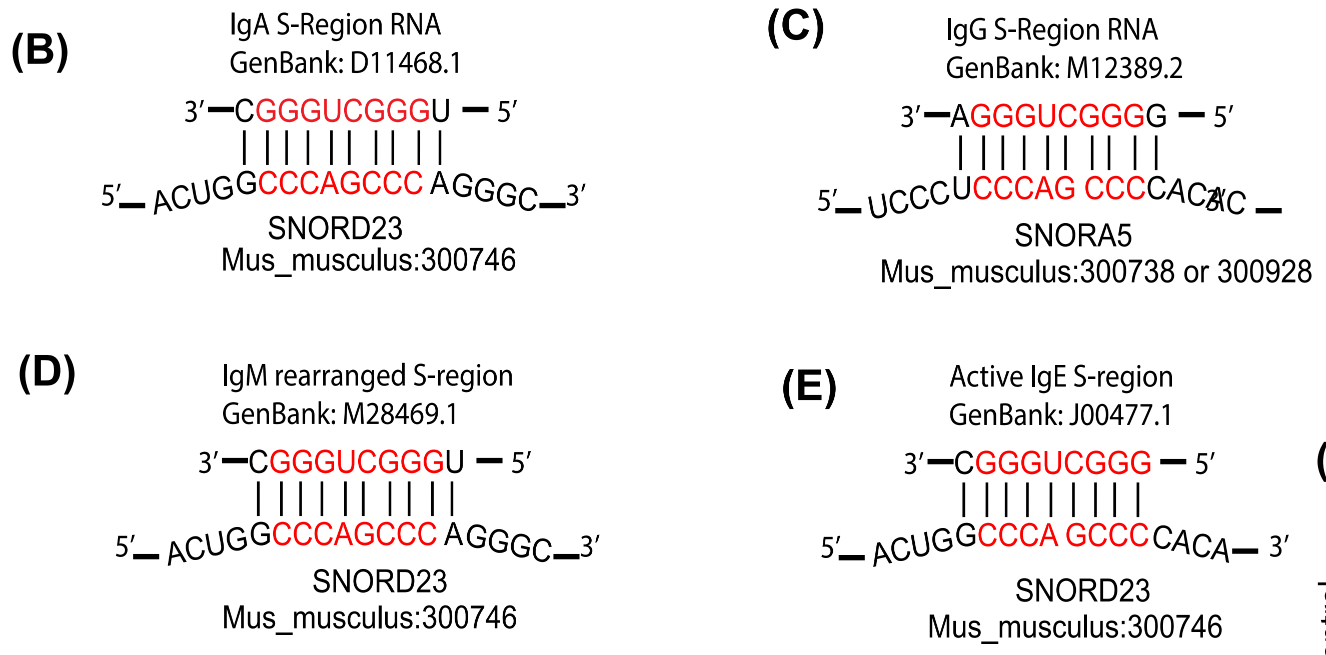

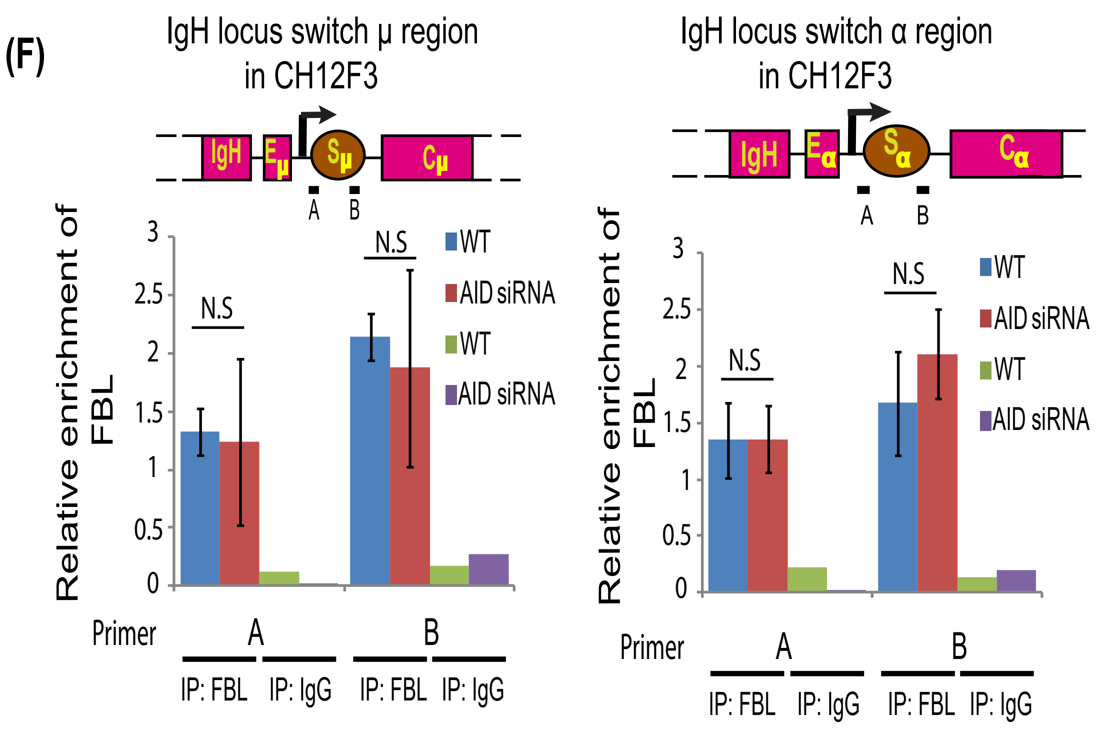

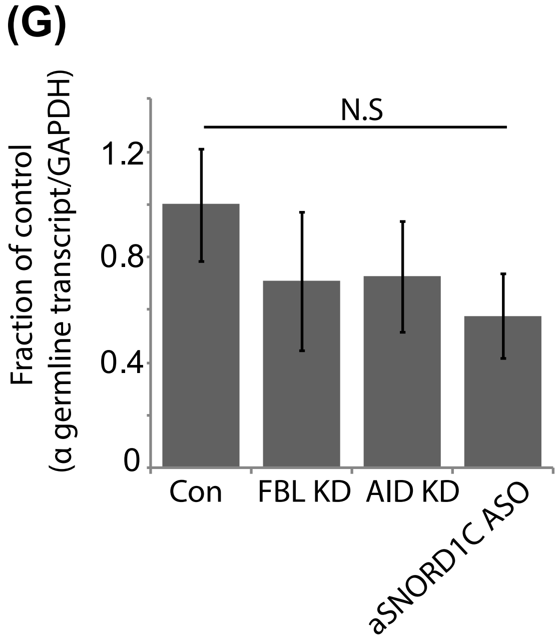

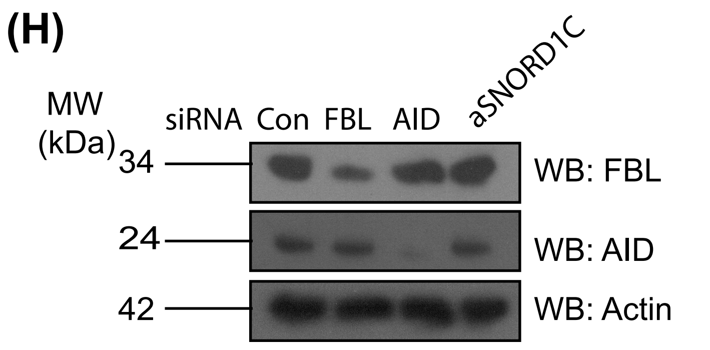

Fig. S7

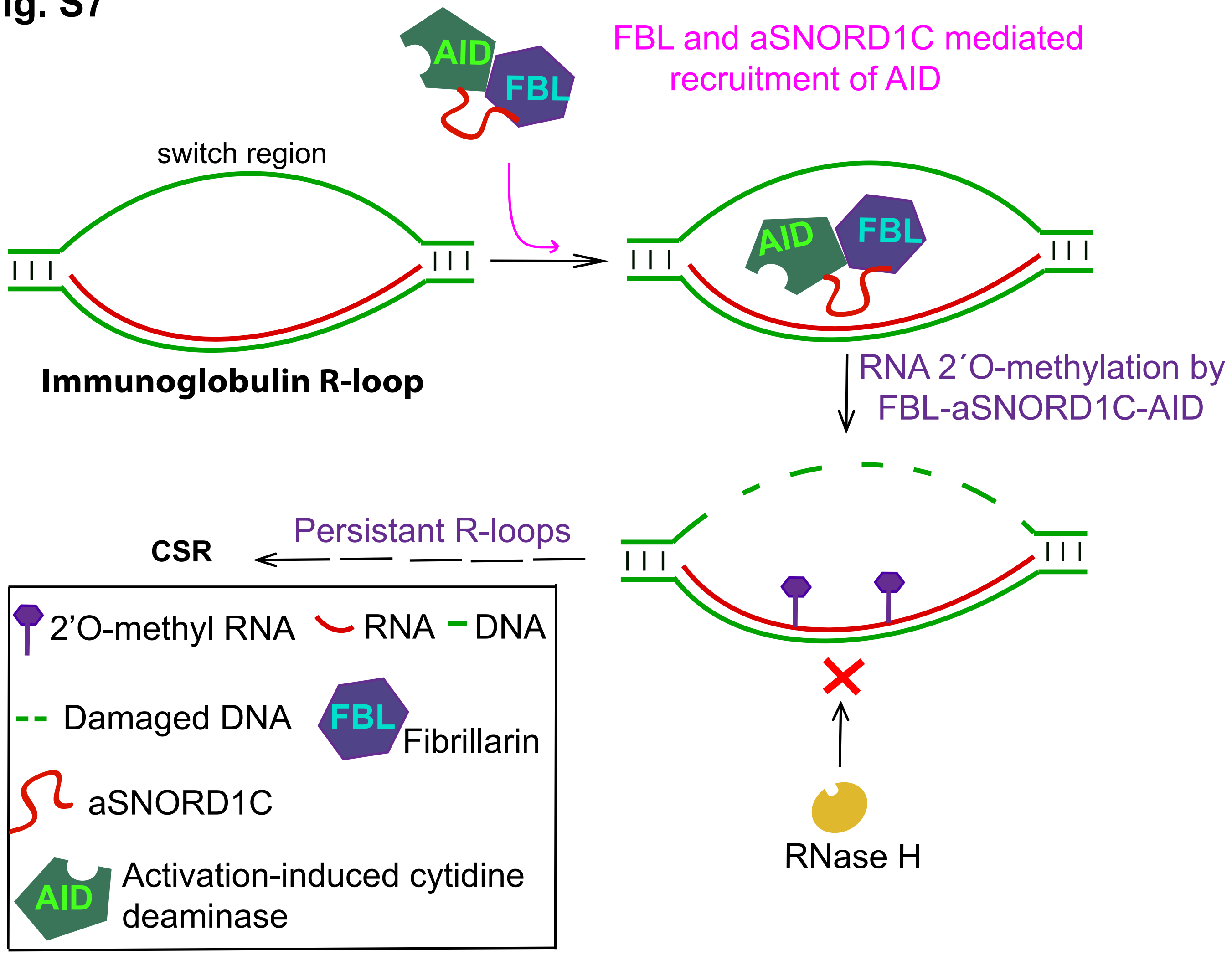

AID
FBL
FBL and aSNORD1C mediated recruitment of AID
switch region
Immunoglobulin R-loop
RNA 2´O-methylation by FBL-aSNORD1C-AID
Persistant R-loops
CSR
RNase H
2’O-methyl RNA
RNA
DNA
Damaged DNA
Fibrillarin
aSNORD1C
Activation-induced cytidine deaminase

Fig. S8

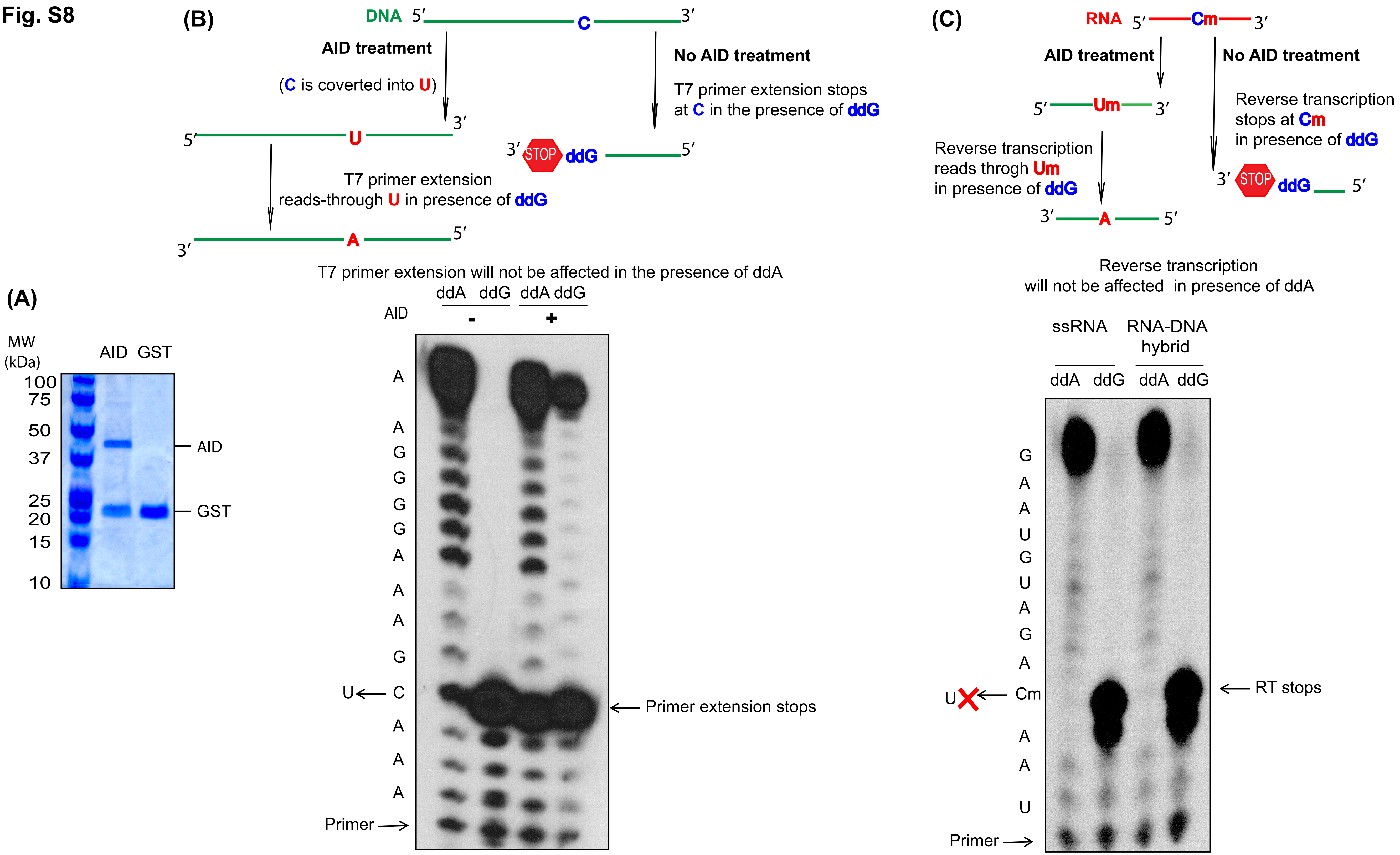

(A)
MW (kDa)
AID GST
100
75
50
37
25
20
15
10
AID
GST
(B)
DNA 5′ C 3′
AID treatment
(C is coverted into U)
3′
5′ U
No AID treatment
T7 primer extension stops at C in the presence of ddG
3′ STOP ddG 5′
T7 primer extension reads-through U in presence of ddG
3′ A 5′
T7 primer extension will not be affected in the presence of ddA
ddA ddG ddA ddG
AID - +
A
A
G
G
G
G
A
A
A
G
U C
A
A
A
Primer
Primer extension stops
(C)
RNA 5′ Cm 3′
AID treatment
No AID treatment
5′ Um 3′
Reverse transcription stops at Cm in presence of ddG
Reverse transcription reads throgh Um in presence of ddG
3′ STOP ddG 5′
3′ A 5′
Reverse transcription will not be affected in presence of ddA
ssRNA
RNA-DNA hybrid
ddA ddG ddA ddG
G
A
A
U
G
U
A
G
A
U Cm
A
A
U
Primer
RT stops
