# Supplementary material for: RNA 2'-O-methylation promotes persistent R-loop formation and AID-mediated IgH class switch recombination
Source: BMC Biol. 2024 Jul 8;22:151. doi: 10.1186/s12915-024-01947-5 (PMC11232215; doi:10.1186/s12915-024-01947-5)
Supplement: Supplementary file 2 — Additional file 2: Table S1. Oligonucleotides used in this study [78–81]. [file 12915_2024_1947_MOESM2_ESM.docx]

**Additional file 2 Table S1: Oligonucleotides used in this study**

| **Oligonucleotides** | **Sense (5′-3′)** | | **Antisense (5′-3′)** |
| --- | --- | --- | --- |
| **qPCR methyltransferase knockdown** | | | |
| CMTR1 | GCGAGCAGAAATTCCTCCTG | | CCCCTCCCCTTTCAGTTCAT |
| FTSJ2 | AATTCCTCCATCGGCTCACA | | TGGATGACTGGCTTGTTCCT |
| FTSJ3 | GACCTCGTTCCAATCAAGCC | | AAGCGTCATGGACCCAACTA |
| FBL | CAGGAGCCAAGGTGCTCTAC | | GCGATAAGCATGCGGTATTT |
| GAPDH | AGGTCGGTGTGAACGGATTTG | | TGTAGACCATGTAGTTGAGGTCA |
| Actin | TGCGTGACATCAAAGAGAAG | | CGGATGTCAACGTCACACTT |
| unspliced αGLTF | GATTTAAGCAGGCCTGGGGTG | | CTAGTTCAGGCCACTCCATG |
| spliced αGLTF | CCAGGCATGGTTGAGATAGAGATAG | | GAGCTGGTGGGAGTGTCAGTG |
| **Semi-quantitative PCR methyltransferase knockdown** | | | |
| RTLP (upstream) | CTGAGCTAGCCTGAACTGGG | | CATCCCATCCCATCCCATCA |
| RTLP (downstream) | GGGATTAGTTAAAATAGGCT | | CATCCCATCCCATCCCATCA |
| RTLP (reverse transcription) | CCATTCAGCTGAGCTCAGCTC | |  |
| **RT primer extension, cDNA library formation using ChIRP** | | | |
| Primer extension | ACCTTACTTTAGCTCAGGTT | | |
| TSO (RT) | GACCTCTTGGCTATGAGCATCCCTCTACGCGATCCAAGCTTCrGrG+G | | |
| TSO (Outer for PCR) | GACCTCTTGGCTATGAGCATCCC | | |
| TSO (Inner for PCR) | CTCTACGCGATCCAAGCTTCG | | |
| **DRIP assay** | | | |
| DRIP Sµ (A)^78^ | CCCGAAGCATTTACAGTGACTTTGTTCATGA | | GATTTGTGAAGCCGTTTTGACCAGAATGTC |
| DRIP Sµ (B)^78^ | CTGCCTACACTGGACTGTTCTGAGC | | CAGCTCACCCCATCTCACCCCATC |
| DRIP Sµ (C)^78^ | GTGTAGGGTGATCTGGACTCAACTGG | | CTTTTCCCAGCTCATCCCGAACC |
| DRIP Sα (A)^16^ | GTTGTGGCTGAGCTGAGCTGG | | CAGTCAGCCCAACTTGGCC |
| DRIP Sα (B) | CTGAGCTAGCCTGAACTGGG | | CATCCCATCCCATCCCATCA |
| DRIP Sα (C)^16^ | CTATCTAAGGCTAAGCCAGGC | | GCTCAGTTAGCCCAGCCCAG |
| DRIP Sγ1 (A) | CTGCTGTGTGGGATCTGCTA | | CCTCTGCCTGAGTTTCCAAG |
| DRIP Sγ1 (B) | CCAGGTGAGAGTACGGGGTA | | TCAGCTCCCATGTAGCTCCT |
| DRIP Sγ1 (C) | ATAGGGCAGCCAGGAGAAAT | | TCCAGGCAGAGCAGTACCTT |
| **AID mediated RNA and DNA deamination** | | | |
| DDR-2 (DNA deamination substrate)^9^ | | AAAGGGGAAAGCAAAGAGGAAAGGTGAGGAGGT | |
| DDR-2P (Primer for the DNA substrate)^9^ | | GAGGAAAGGTGAGGAGGT | |
| RNA2-O′Me (RNA deamination substrate) | | gaauguaugagmcaauagaagagauaaugaauaauagaa | |
| DNA-RNA2-O′Me (*complementary DNA to* RNA2-O′Me)^79^ | | TTC TAT TAT TCA TTA TCT CTT CTA TTG CTC ATA CAT TC | |
| RNA2-O′mP (Primer for the RNA substrate)^79^ | | ttctattattcattatct | |
| **snoRNA qPCR, ASO and LNA probes** | | | |
| U50^80^ | AGTCTATGATGATCCTATCCCG | | ATCTCAGAAGCCAGATCCGTAAGTATGG |
| SNORD1C (human) | TGAGCTGAGGATGATTTA | | TGAGCCTCAGCAAGTCAG |
| SNORD1C (mice) | TCGAGCCTCAGTAAACCATG | | TTGAGCTGAGGATGATTTAA |
| 36B4^81^ | ATCCCTGACGCACCGCCGTGA | | TGCATCTGCTTGGAGCCCACGTT |
| CH12F3 Directional RT | GTTGAGCTGAGGATGATTTA | | GTCGAGCCTCAGTAAACCAT |
| HEK293 Directional RT | GCTGAGCTGAGGATGATTTA | | GCTGAGCTGAGGATGATTTA |
| ASO1 | T*A*T*C*C*C*T*G*T*C*T*G*A*A*A*T*G*A*C*A | | |
| ASO2 | G*A*C*A*C*T*T*T*T*G*T*G*A*A*G*A*G*T*C*A | | |
| ASO Control | A*T*T*A*T*A*C*A*A*T*C*T*C*G*C*G*A*C*G*T | | |
| (1) LNA U1581 | mC*mA*mC*mU*C*C*C*A*A*C*C*A*T*T*T*C*A*G*mC*mA*mA*mG | | |
| (2) LNA U1581 | mC*mC*mC*mA*C*T*C*C*C*A*A*C*C*A*T*T*T*C*mA*mG*mC*mA | | |
| (1) LNA IgA | mC*mC*mC*mC*A*G*G*T*C*A*C*A*T*T*C*A*T*C*mG*mU*mG*mC | | |
| (2) LNA IgA | mC*mA*mG*mG*T*C*A*C*A*T*T*C*A*T*C*G*T*mG*mC*mC*mG | | |
