# Supplementary material for: RNA 2'-O-methylation promotes persistent R-loop formation and AID-mediated IgH class switch recombination
Source: BMC Biol. 2024 Jul 8;22:151. doi: 10.1186/s12915-024-01947-5 (PMC11232215; doi:10.1186/s12915-024-01947-5)

Fig. 1

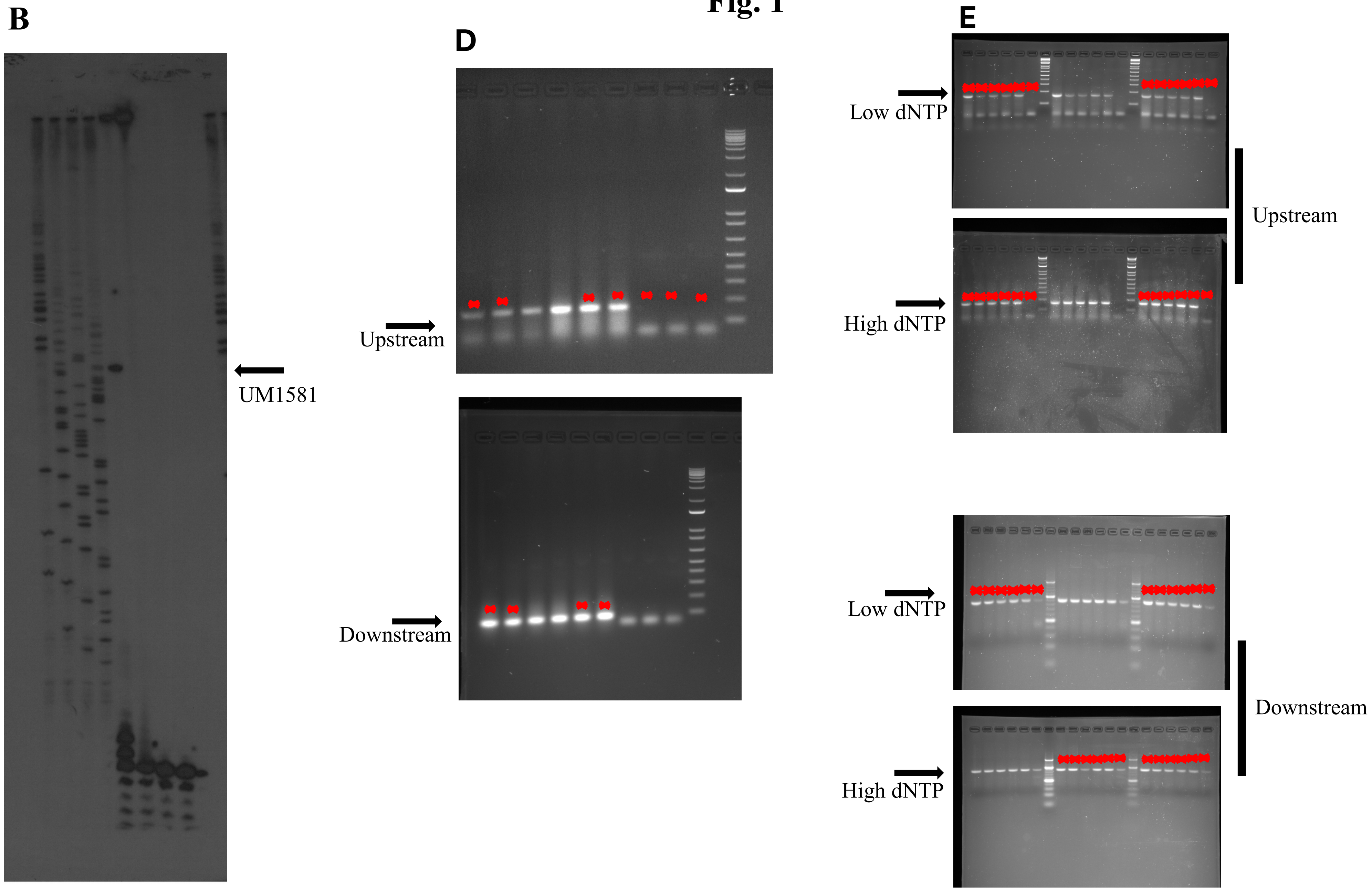
B
UM1581
D
Upstream
Downstream
E
Low dNTP
High dNTP
Upstream
Low dNTP
High dNTP
Downstream

Fig. 3

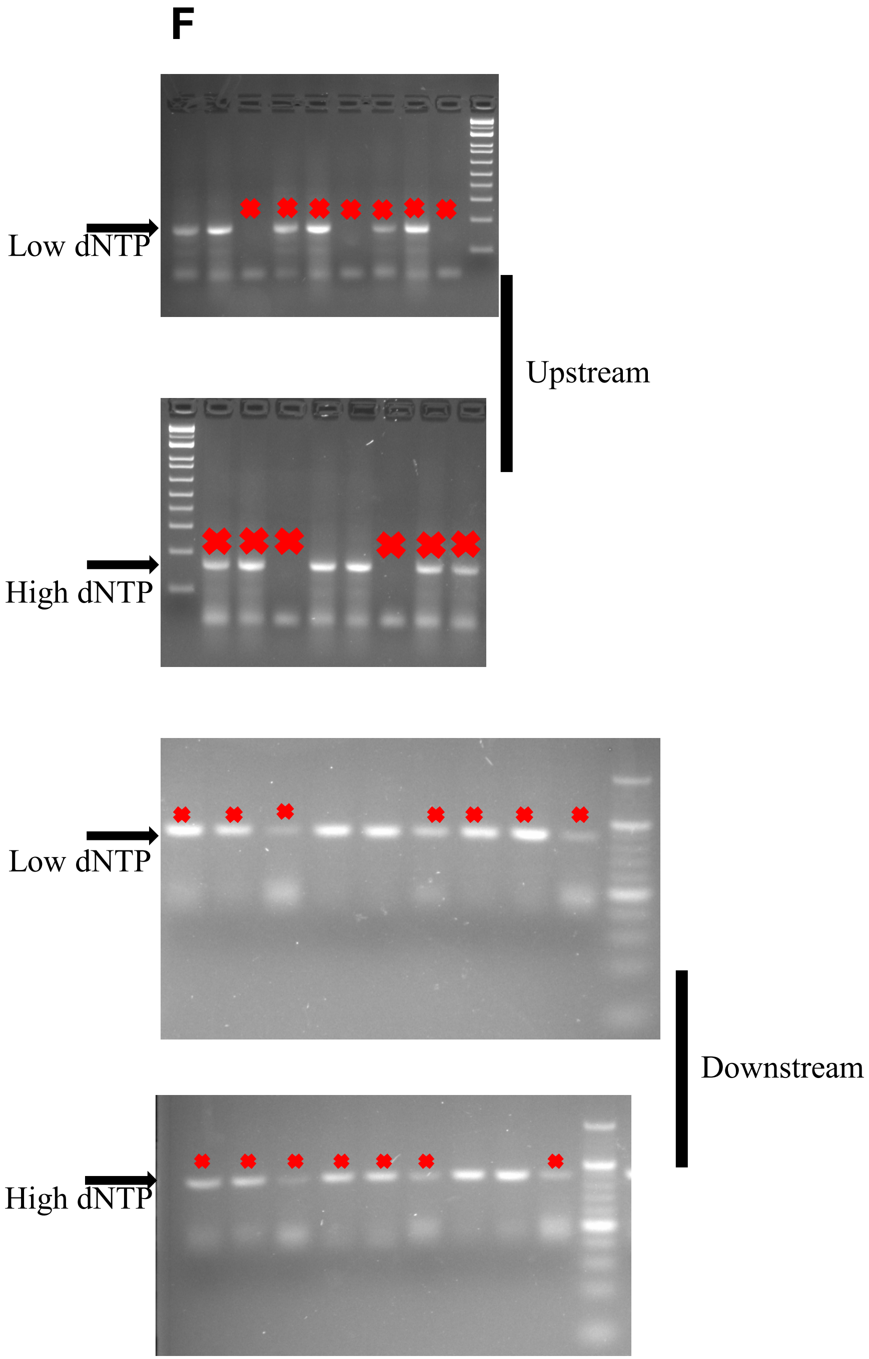

Fig. 4

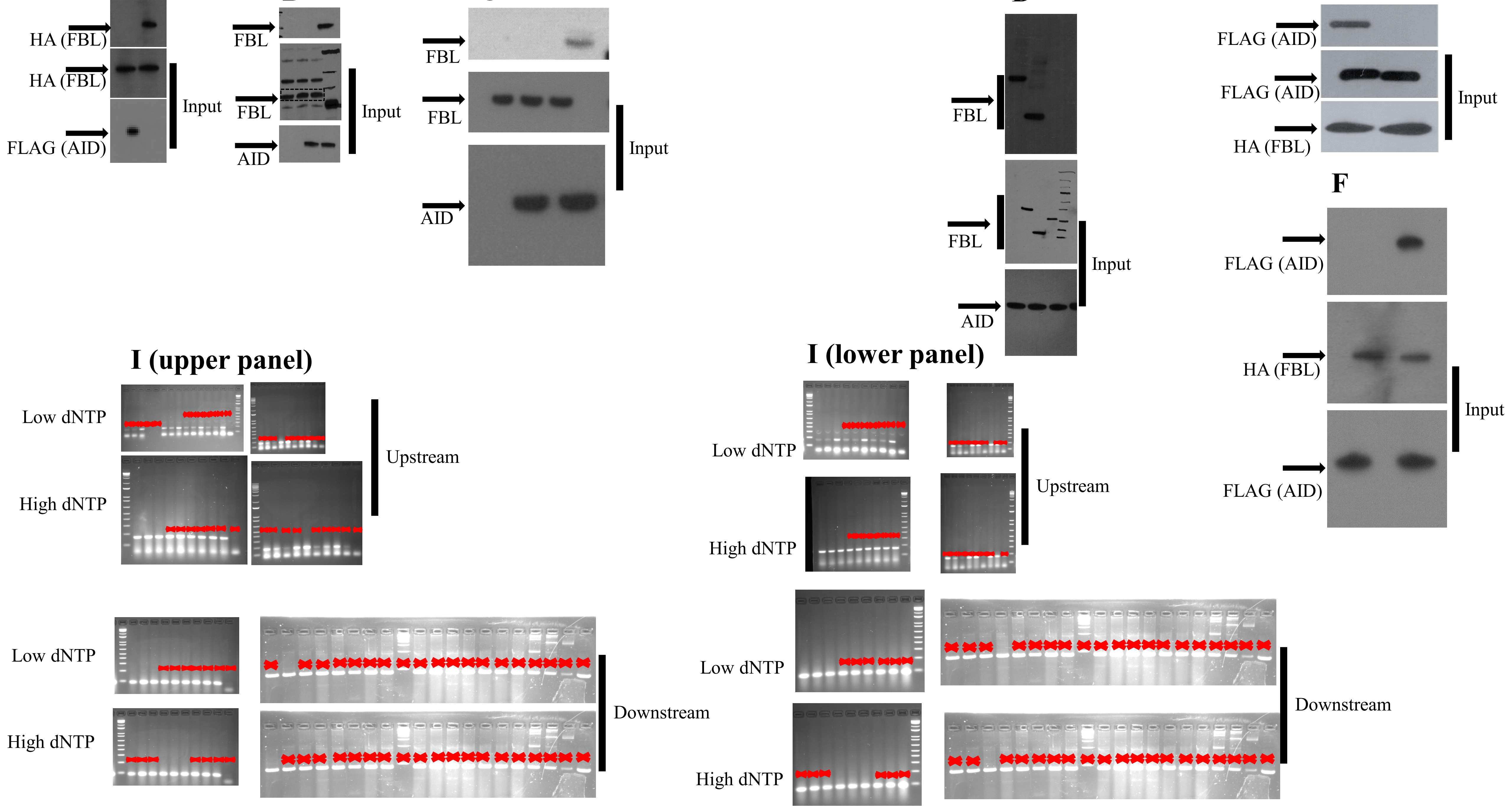
A
HA (FBL)
HA (FBL)
FLAG (AID)
Input
B
FBL
FBL
AID
Input
C
FBL
FBL
AID
Input
D
FBL
FBL
AID
Input
E
FLAG (AID)
FLAG (AID)
HA (FBL)
Input
F
FLAG (AID)
HA (FBL)
FLAG (AID)
Input
I (upper panel)
Low dNTP
High dNTP
Upstream
Low dNTP
High dNTP
Downstream
I (lower panel)
Low dNTP
High dNTP
Upstream
Low dNTP
High dNTP
Downstream

Fig. 5

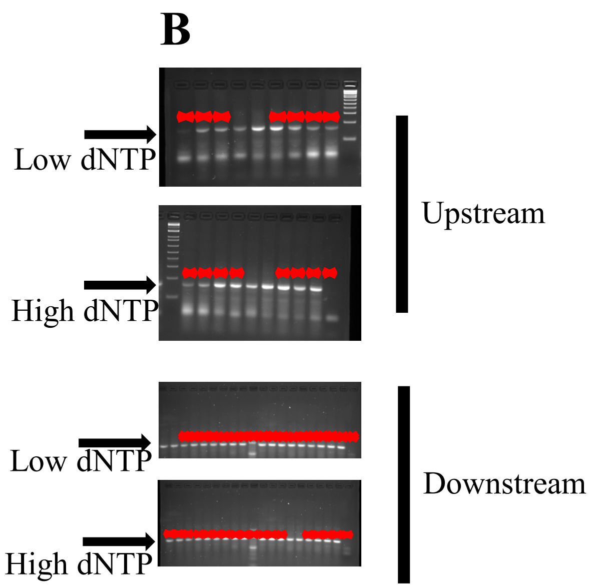

Additional file 1: Fig. S1

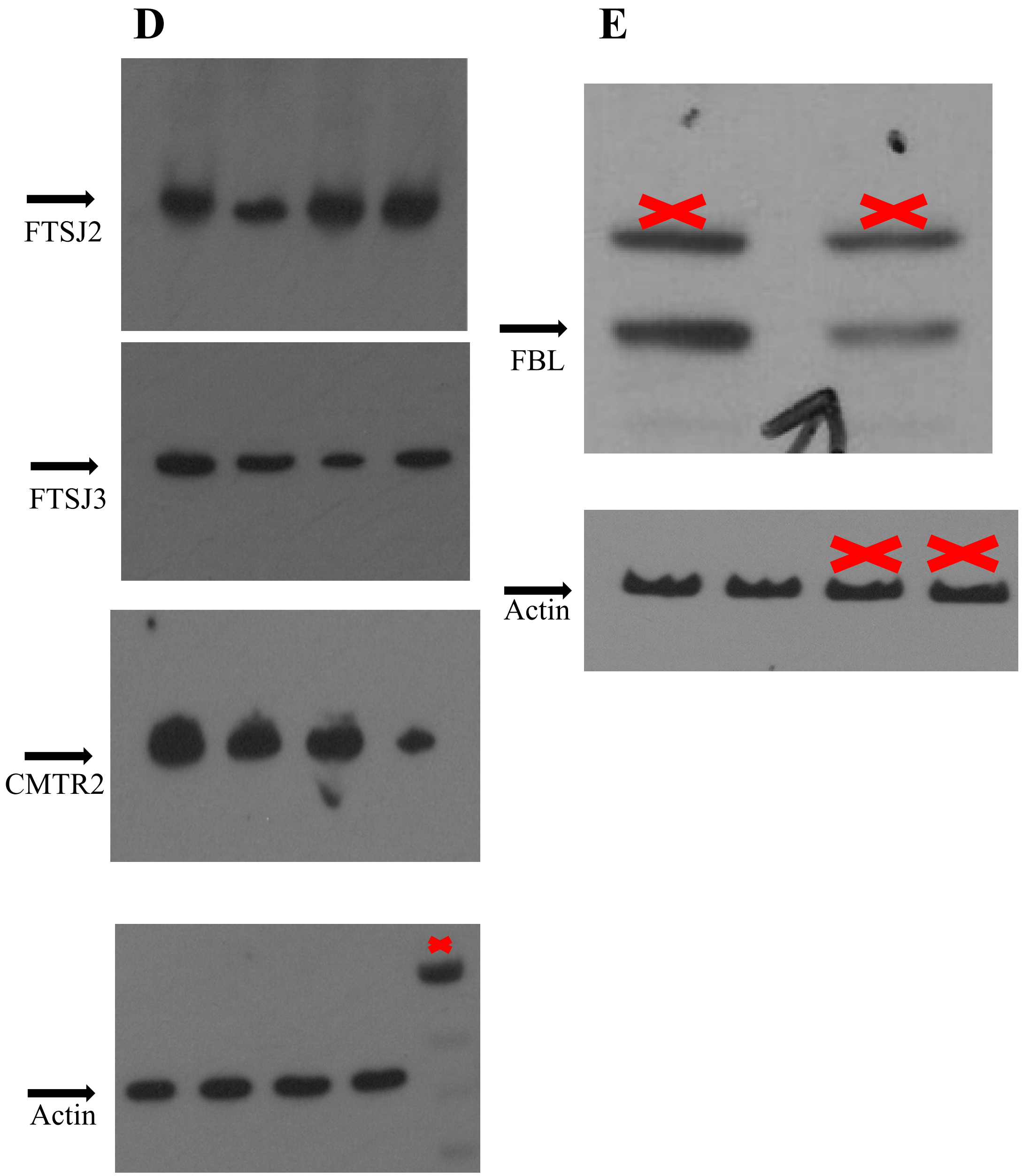

Additional file 1: Fig. S2

A

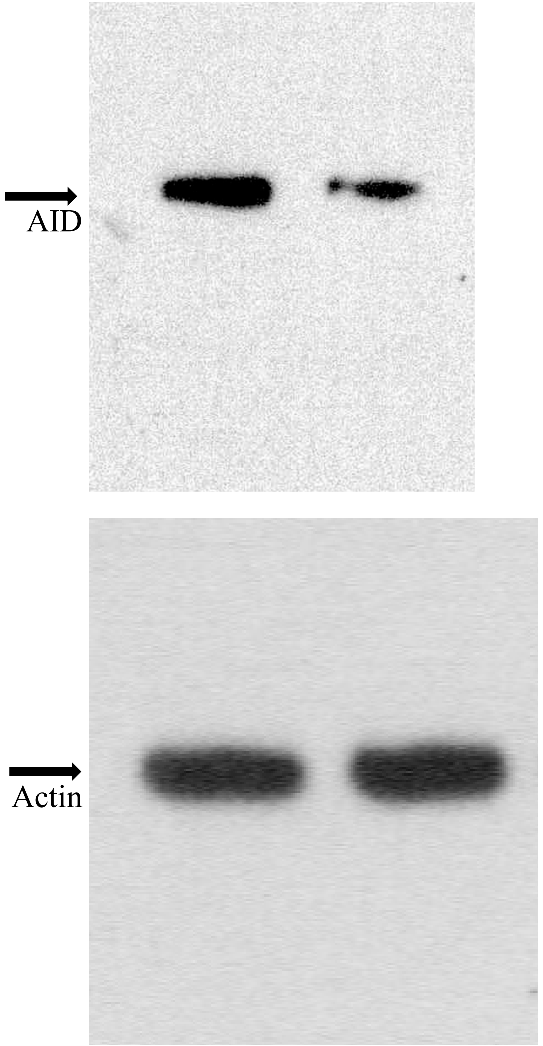

Additional file 1: Fig. S4

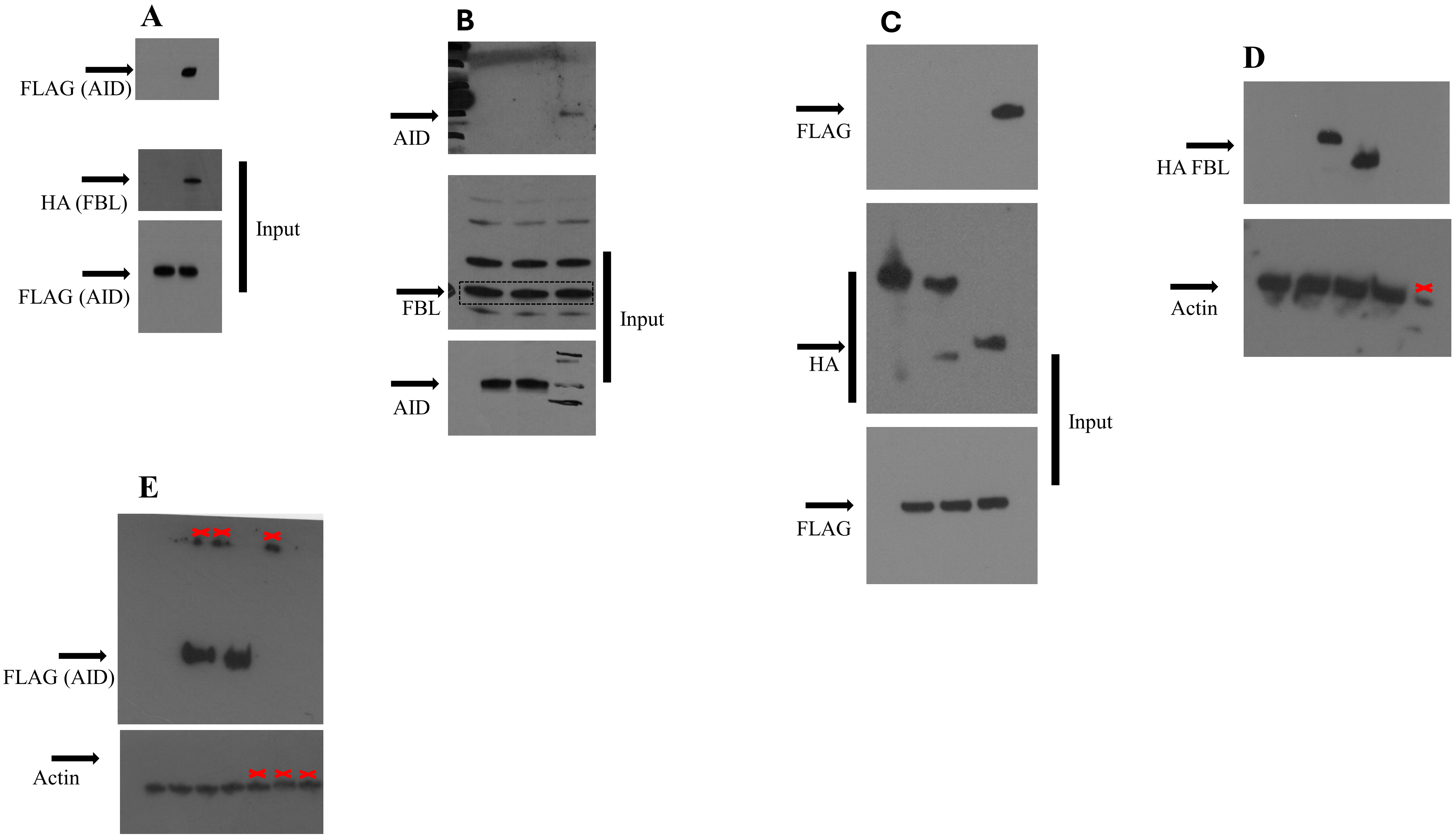

Additional file 1: Fig. S5

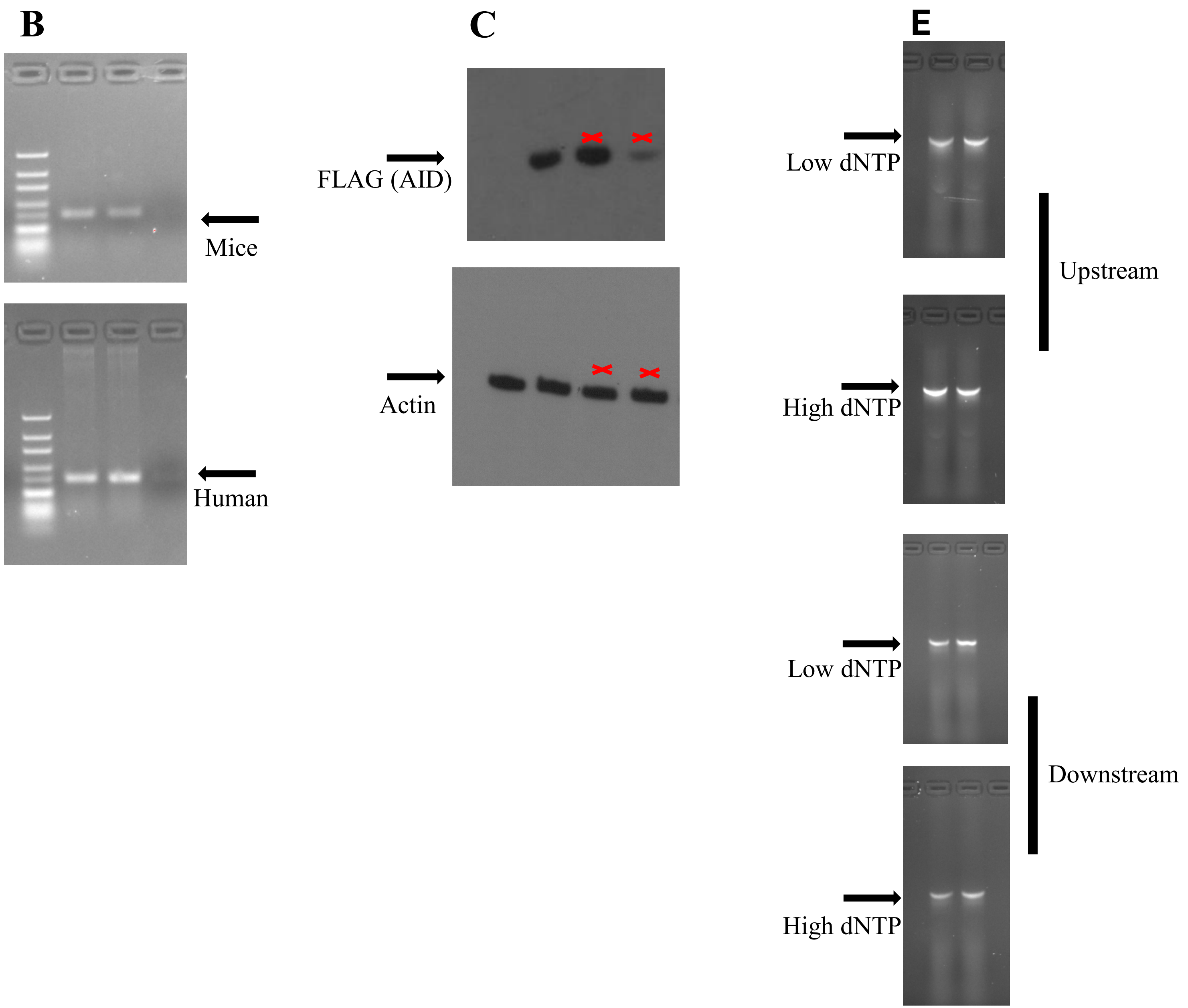

Additional file 1: Fig. S6

H

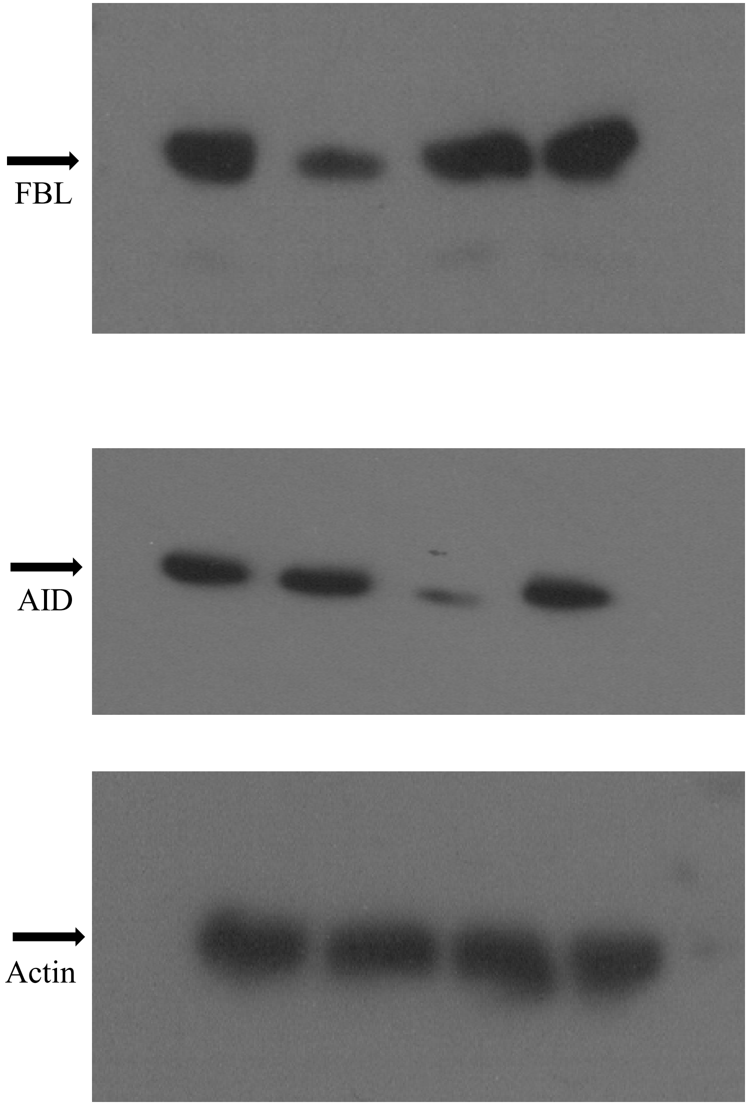

Additional file 1: Fig. S8

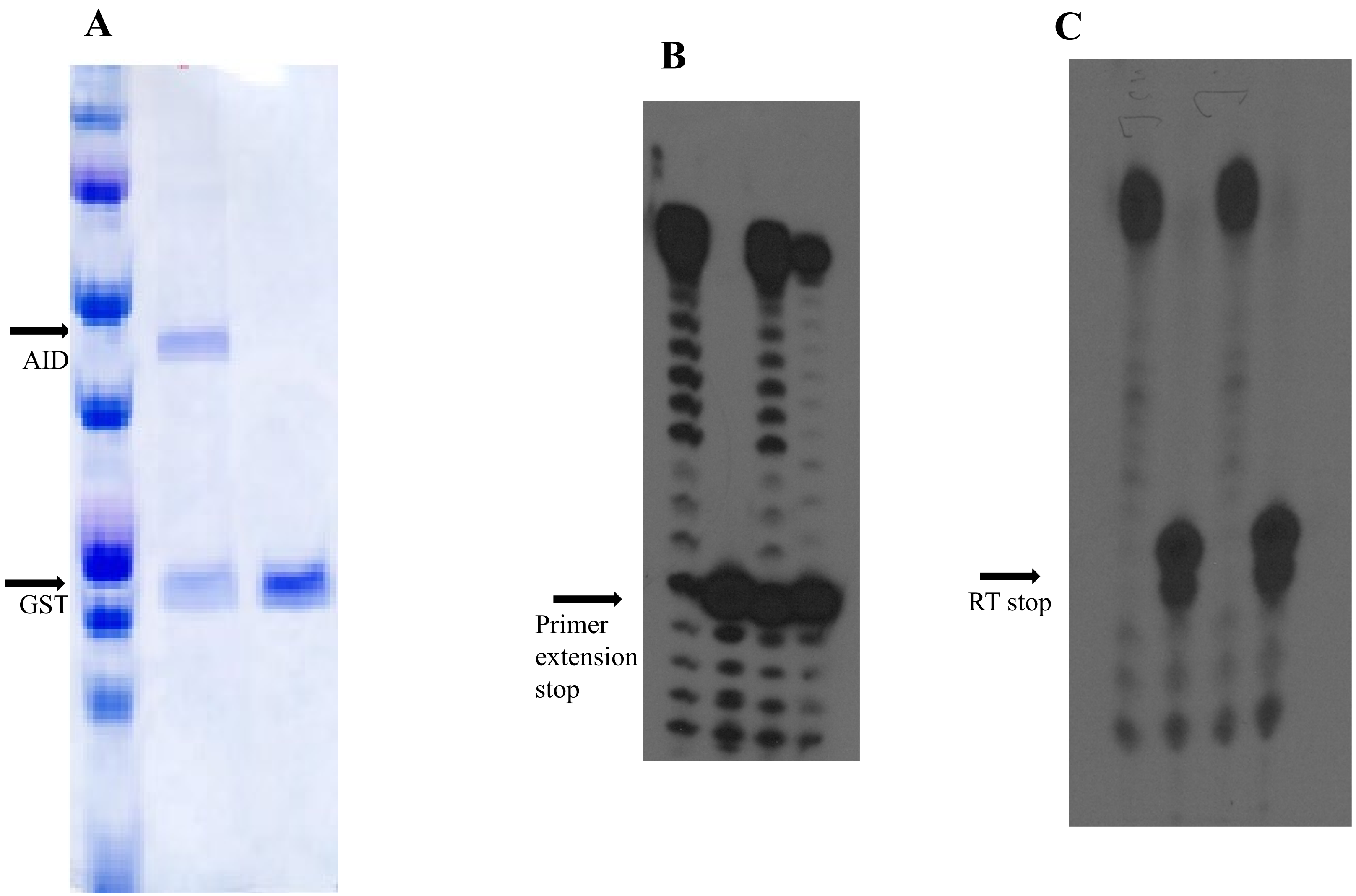

Supplement: Supplementary file 3 — Additional file 3: Raw data. [file 12915_2024_1947_MOESM3_ESM.pdf]
